# Genome-Wide Identification of Chromatin Transitional Regions Reveals Diverse Mechanisms Defining the Boundary of Facultative Heterochromatin

**DOI:** 10.1371/journal.pone.0067156

**Published:** 2013-06-28

**Authors:** Guangyao Li, Lei Zhou

**Affiliations:** Graduate Program in Genetics and Genomics, University of Florida Genetics Institute; Department of Molecular Genetics and Microbiology & University of Florida Shands Cancer Center, College of Medicine, University of Florida. Gainesville, Florida, United States of America; Florida State University, United States of America

## Abstract

Due to the self-propagating nature of the heterochromatic modification H3K27me3, chromatin barrier activities are required to demarcate the boundary and prevent it from encroaching into euchromatic regions. Studies in *Drosophila* and vertebrate systems have revealed several important chromatin barrier elements and their respective binding factors. However, epigenomic data indicate that the binding of these factors are not exclusive to chromatin boundaries. To gain a comprehensive understanding of facultative heterochromatin boundaries, we developed a two-tiered method to identify the Chromatin Transitional Region (CTR), i.e. the nucleosomal region that shows the greatest transition rate of the H3K27me3 modification as revealed by ChIP-Seq. This approach was applied to identify CTRs in *Drosophila* S2 cells and human HeLa cells. Although many insulator proteins have been characterized in *Drosophila*, less than half of the CTRs in S2 cells are associated with known insulator proteins, indicating unknown mechanisms remain to be characterized. Our analysis also revealed that the peak binding of insulator proteins are usually 1–2 nucleosomes away from the CTR. Comparison of CTR-associated insulator protein binding sites vs. those in heterochromatic region revealed that boundary-associated binding sites are distinctively flanked by nucleosome destabilizing sequences, which correlates with significant decreased nucleosome density and increased binding intensities of co-factors. Interestingly, several subgroups of boundaries have enhanced H3.3 incorporation but reduced nucleosome turnover rate. Our genome-wide study reveals that diverse mechanisms are employed to define the boundaries of facultative heterochromatin. In both *Drosophila* and mammalian systems, only a small fraction of insulator protein binding sites co-localize with H3K27me3 boundaries. However, boundary-associated insulator binding sites are distinctively flanked by nucleosome destabilizing sequences, which correlates with significantly decreased nucleosome density and increased binding of co-factors.

## Introduction

Site-specific formation of facultative heterochromatin, mediated by PcG (Polycomb group) proteins, plays a fundamentally important role in controlling cellular differentiation and in defining the property of differentiated cells. The suppressive histone modification mark, H3K27me3, is catalyzed by Polycomb repressive complex 2 (PRC2). This suppressive modification has strong affinity to, and is usually bound by, Polycomb repressive complex 1 (PRC1). The interaction between PRC1 and PRC2 leads to the propensity to spread this suppressive histone modification until it is antagonized (reviewed in [1], [2]). Although certain strong promoters of active genes can prevent the formation of facultative heterochromatin [3], under many circumstances, specialized DNA elements called chromatin barriers or barrier insulators are needed to demarcate the boundary of facultative heterochromatin (reviewed in [4]).

Insulators, such as the *gypsy* insulator, were originally identified for their enhancer-blocking activity, i.e. blocking the interaction between the enhancer and promoter when placed in-between [5]. Later, it was revealed that most of them also have barrier activity [6], [7], i.e. blocking the propagation of repressive histone modifications. It was not clear whether the two activities are separable until the characterization of the cHS4 insulator in the chicken β-globin locus. The complete cHS4 has both enhancer-blocking and barrier activity. However, a series of mechanistic studies indicated that the two activities are separable and carried out by distinct DNA elements. The enhancer-blocking activity of cHS4 is mediated by CTCF, while its barrier activity against heterochromatin formation requires a binding site for USF1 (Upstream Stimulatory Factor 1). Binding of USF1 to cHS4 recruits chromatin-modifying enzymes that catalyze histone modifications incompatible with heterochromatin formation, thus preventing the propagation of suppressive histone modification [8], [9].

Recently, a novel chromatin barrier that lacks any detectable enhancer-blocking function has also been identified in *Drosophila* [10]. This ∼200 bp element is located at the left boundary of IRER (Irradiation Responsive Enhancer Region), a 33 kb intergenic regulatory region controlling stress-induced expression of multiple pro-apoptotic genes [11]. When tested in transgenic animals, ILB (IRER Left Boundary) is fully capable of blocking the propagation of H3K27me3 initiated by a strong Polycomb response element (PRE) [10]. The chromatin barrier function of ILB is evolutionarily conserved. When tested in a vertebrate system, it blocked heterochromatin propagation as effectively as the cHS4 [10]. Although many insulator/boundary-associated proteins have been characterized in *Drosophila*, including Su(Hw), dCTCF, BEAF-32, GAF, CP190 and Mod(mdg4) (reviewed in [12]), none of those was found associated with ILB. The presence and prevalence of novel boundary-setting mechanisms were also implicated by epigenomic studies conducted in *Drosophila* and mammalian systems, which revealed that the majority of H3K27me3 boundaries are not associated with characterized insulator proteins.

Although lots of efforts have been directed towards partitioning the genome into large domains based on multiple histone modifications [13], [14] or protein binding profiles [15], there is much less focus on understanding how individual repressive histone modification is demarcated by chromatin barrier elements. To gain a comprehensive understanding of boundaries of facultative heterochromatin, we developed a novel bioinformatics approach to identify the chromatin transitional regions (CTRs). We reasoned that if the propagation of heterochromatin formation is stopped by a counter-acting mechanism as revealed by the models proposed by Felsenfeld and colleagues [4], then the boundary of the facultative heterochromatin should manifest as a rapid transitional region where the level of H3K27me3 shows dramatic changes. Using a two-tiered approach, we demonstrated that it is feasible to identify the CTRs based on H3K27me3 ChIP-Seq data from both *Drosophila* and mammalian cell lines. By locating CTRs to single nucleosome resolution, we found that CTRs are usually 1–2 nucleosomes away from the binding sites of known insulator/boundary-associated factors. However, the majority of CTRs are not associated with any known insulator proteins. Conversely, only a small portion of insulator protein binding sites are associated with CTRs. Comparing insulator protein bindings associated with CTRs vs. those in H3K27me3-enriched regions revealed interesting distinctions in co-factor binding as well as in DNA sequences flanking the binding sites. Overall, our analysis suggests that diverse mechanisms can be employed to establish the boundaries of facultative heterochromatin.

## Results

### Localize the Chromatin Transitional Regions (CTRs) Based on H3K27me3 ChIP-Seq Data

At the time of our study, several methodologies, such as SICER [16] and RSEG [[17], have been developed to analyze genomic profiles of H3K27me3, the signature marker of facultative heterochromatin. Most of these methodologies focus on identifying broad domains enriched for a particular histone modification. Although these methodologies are very useful for identifying H3K27me3-enriched regions, they were not designed for the purpose of specifying the boundary of facultative heterochromatin. The fact that there is a lack of experimentally verified data set of H3K27me3 boundaries also prevented objective comparison of these methodologies.

*Drosophila melanogaster* provides the best system for studying the boundaries of facultative heterochromatin. Several insulator proteins, such as Su(Hw) [18], BEAF-32 [19], and dCTCF [20], have been very well characterized in *Drosophila*. The genome-wide binding profiles for these proteins, as well as many other genomic and epigenomic information, are available for the *Drosophila* Schneider 2 (S2) cells due to the efforts of the modENCODE project [21] and many other individual labs. Taking the advantage of these data, we generated an empirical set of chromatin transitional regions for H3K27me3 (Figure S1 in File S1). In essence, we selected regions where clear changes in H3K27me3 enrichment, as revealed by ChIP-Seq, were accompanied by experimentally verified binding of the insulator proteins and their respective co-factors (such as CP190).

**Figure 1:**
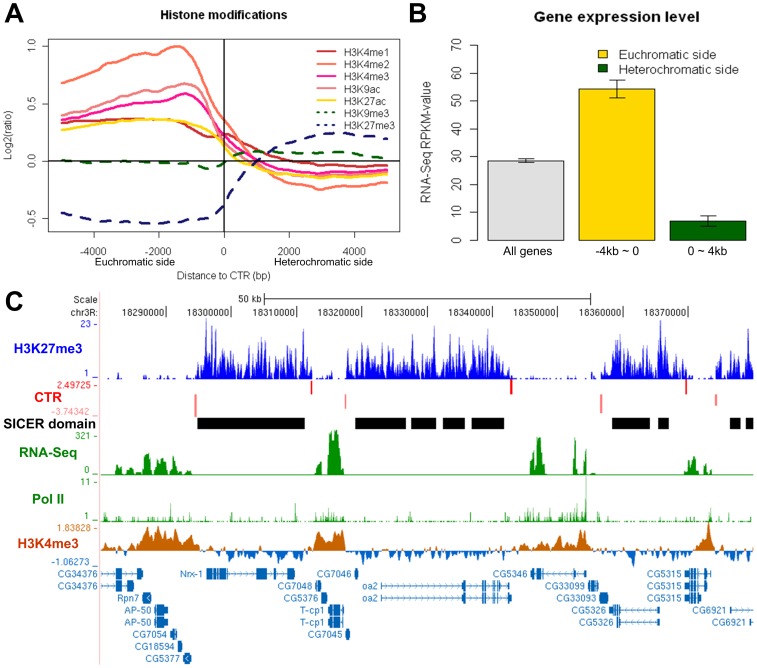
Histone modifications and gene expression levels on the euchromatic vs. heterochromatic side of the CTRs in *Drosophila* S2 cell line. (A) Enrichment levels of active (solid lines) and repressive (dashed lines) histone modifications around the H3K27me3 CTRs identified in S2 cells. Negative and positive distances indicate euchromatic and heterochromatic sides of the identified CTRs, respectively. (B) Expression levels of genes on the euchromatic or heterochromatic side of CTRs. Barplots represent Mean±SE for all genes (grey), genes within the 4 kb region on the euchromatic side (yellow) or the heterochromatic side (green) of CTRs. The expression levels for genes on euchromatic side of CTRs are significantly greater than those of the genes on the heterochromatic side (p<2.2E-16, Wilcoxon rank sum test). (C) An example of 7 CTRs (red bars) predicted by CTRICS. Bar height reflects T-score, top and bottom rows denotes the orientation of the CTRs. The panel below CTR shows H3K27me3 domains called by SICER. RNA-Seq signal, RNA Pol II binding, as well as active histone modification (H3K4me3) are depleted in heterochromatic regions which have high H3K27me3, while they are enriched in euchromatic regions.

Testing of the two popular H3K27me3 enrichment-calling algorithms with this empirical H3K27me3 boundary data set revealed inconsistency in precisely defining the transition region. We noticed that the enrichment-calling algorithms such as SICER is sensitive to the fluctuation of H3K27me3 enrichment levels in continuous facultative heterochromatin regions, and consequently predicts many “extra” boundaries in areas where the enrichment level of H3K27me3 fluctuated (Figure 1C, Figure S2 in File S1). On the other hand, methodology such as RSEG, which based on the two-state hidden Markov model and provided specific boundary calling function, seems to miss some putative boundaries in our empirical data set (Fig. S2). It is worth noting that RSEG also failed to predict a boundary at the ILB locus, which has been experimentally verified to function as chromatin barrier against Polycomb group (PcG)-mediated spreading of H3K27me3 [10].

To pinpoint the location of CTR, we developed a two-tiered analysis methodology called CTRICS (Chromatin Transitional Regions Inference from ChIP-Seq) (see Methods for detail). First, the existence of a transition was detected by comparing the enrichment of H3K27me3 in relatively large genomic intervals (4 kb). The relatively large interval helps to minimize the false positives due to the fluctuation of H3K27me3 enrichment levels in facultative heterochromatin regions. After a transitional event has been identified, a secondary analysis is performed with short intervals to identify a 200bp region where the enrichment of H3K27me3 displays the most significant change. The number of CTRs identified by CTRICS is comparable to the boundaries identified by RSEG, and both are much less when compared with the boundaries predicted by SICER (Figure S2A in File S1). The majority of CTRs we identified overlap (i.e. within 2 kb) with the boundaries predicted by RSEG (Figure S2A in File S1). However, unlike RSEG, our method was able to identify more putative boundaries in our empirical data set (Figure S2B in File S1) as well as the ILB. Visual inspection indicated that some of the CTRs identified by CTRICS, but missed by RSEG can be corroborated with other evidences such as RNA-Seq or H3K4me3 data (Figure S2C in File S1). Thus we resorted to use CTRICS for genome-wide analysis of CTRs in S2 cells.

### Genome-wide Identification of CTRs in S2 Cells

Applying CTRICS to the H3K27me3 ChIP-Seq dataset derived from the *Drosophila* S2 cell line [22] identified a total of 2082 CTRs. From sequencing depth analysis, we noticed that the H3K27me3 ChIP-Seq dataset, with a total of ∼2.8 million uniquely mapped reads, had already reached saturation plateau for CTR detection (Figure S3 in File S1).

Since CTRs define the boundaries between repressive facultative heterochromatin and accessible euchromatin, the active and repressive histone marks should have contrasting patterns around CTRs. Indeed, active histone marks, such as H3K4me1, H3K4me2, H3K4me3, H3K9ac, and H3K27ac, are enriched on the euchromatic side of CTRs, while depleted on the heterochromatic side (Figure 1A). We noticed that the enrichment levels of H3K9me3, which mostly associate with constitutive heterochromatin in centromeric and telomeric regions [23], do not change significantly around the identified CTRs. This indicated that the CTRs we identified are specific to facultative heterochromatin. Although the two repressive histone marks overlap at some loci [10], [24], [25], their global localizations are largely independent of each other. Our analysis also suggested that in most loci, the change of H3K27me3 level at the boundary was not associated with significant changes in H3K9me3.

In addition, we reasoned that genes locate on the heterochromatic sides of CTRs should in general be repressed compared to those on the euchromatic side. When the expression profile was evaluated using a companying RNA-Seq dataset from S2 cells [22], the difference was indeed obvious for genes on different sides of the CTRs. Compared with the global average, genes whose entire transcribed regions locate within the 4 kb regions on the euchromatic side of CTRs had significantly higher level of expression, whereas genes on the heterochromatic sides were significantly repressed (Figure 1B). Corresponding with the difference in gene expression levels, the binding of Pol II as well as active histone modification H3K4me3 show specific enrichment on the euchromatic side of CTRs (Figure 1C). These evidences all support that the CTRs identified by our method indeed are sharp boundaries interface H3K27me3-enriched and depleted regions.

### The Spatial Relationships Between CTRs and Known Boundary-setting Proteins

**Figure 2:**
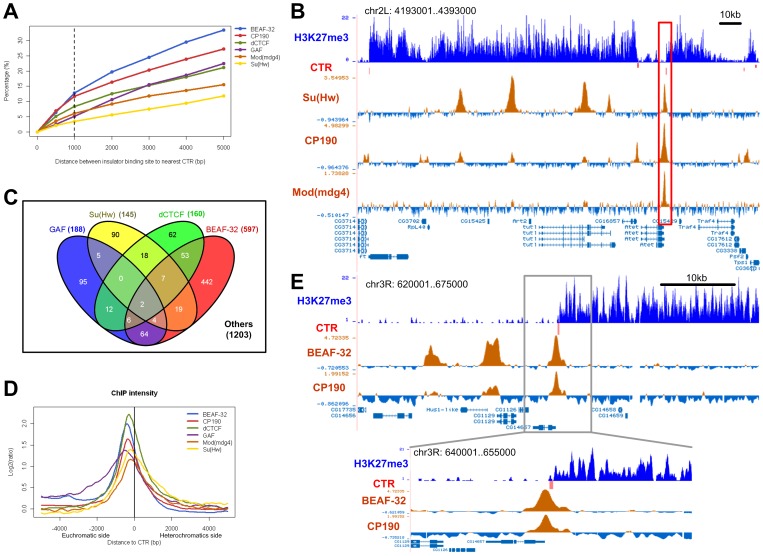
CTRs and the known insulator proteins in *Drosophila* S2 cell line. (A) Percentages of insulator protein binding sites are associated with a CTR. The x-axis shows the distance between insulator protein binding site and the nearest CTR, and y-axis shows the percentage of binding sites that are within a certain distance from the nearest CTR. The dashed line indicates the distance cutoff of 1 kb, which is used for association analysis. (B) A 200 kb region on chromosome 2L as an example. There are five Su(Hw) binding sites in this region, one is associated with a CTR (red bar, highlighted region), the others locate in regions enriched for H3K27me3. The intensities of co-factors (CP190, Mod(mdg4)) are relatively high at the CTR-associated binding site, and lower at the binding sites in the H3K27me3-enriched region. (C) Venn diagram shows the number of CTRs that are associated with four insulator proteins. Note that more than half (1203/2082) of the CTRs are not associated with any of the four insulator proteins. (D) Enrichment of insulator proteins in the +5 kb region around corresponding CTRs. The negative and positive distances also indicate the euchromatic and heterochromatic side of CTR, respectively. (E) An example illustrates the relative positions of a predicted CTR and the binding profiles of BEAF-32 and CP190. The peaks of the binding sites locate on the euchromatic side of the CTR, and the distance between the peaks of binding sites and the CTR midpoint is about 400 bp.

The global binding profiles of the major insulator proteins Su(Hw), BEAF-32, dCTCF, GAF, and their important co-factors (such as CP190 and Mod(mdg4)) are available for the S2 cells. Comparison of H3K27me3 CTRs and the binding profiles indicated that less than 15% of the insulator proteins binding sites are within 1 kb of the identified CTRs (Figure 2A). The majority of the binding sites of these known insulator proteins are not in close association with CTRs. For instance, many (∼49%) Su(Hw) binding sites are found in continuous H3K27me3 domains (Figure 2B).

Conversely, less than half (∼42%) of the H3K27me3 CTRs are associated with any of the four DNA-binding insulator proteins, i.e. located within 1 kb (Figure 2C). However, for those that do associate with a binding site for the insulator proteins, the binding site is always preferentially located at the euchromatic side of the CTR (Figure 2D,E), which agrees very well with a recent genome-wide study of chromatin boundary elements conducted in human CD4^+^ cells [26]. When the intensity of these proteins were plotted, the peaks of insulator binding is located at about 2 nucleosomes (200–600 bp) away from the CTR (Figure 2D). Very similar spatial relationship between CTRs and insulator protein binding was observed for BEAF-32, Su(Hw), and dCTCF. Compared with these insulator proteins, the spatial relationship between GAF binding and the correlated CTRs was somewhat different, with the enrichment region more spread out and the peak of binding intensity about 1 more nucleosome away from the CTR (Figure 2D). Figure 2E illustrates a CTR as an example, it is associated with BEAF-32 and CP190, and the peaks of both protein binding sites are located on the euchromatic side of this CTR, with about 400 bp between the peaks of binding and the CTR.

### The Diversity of Facultative Heterochromatin Boundaries

**Figure 3:**
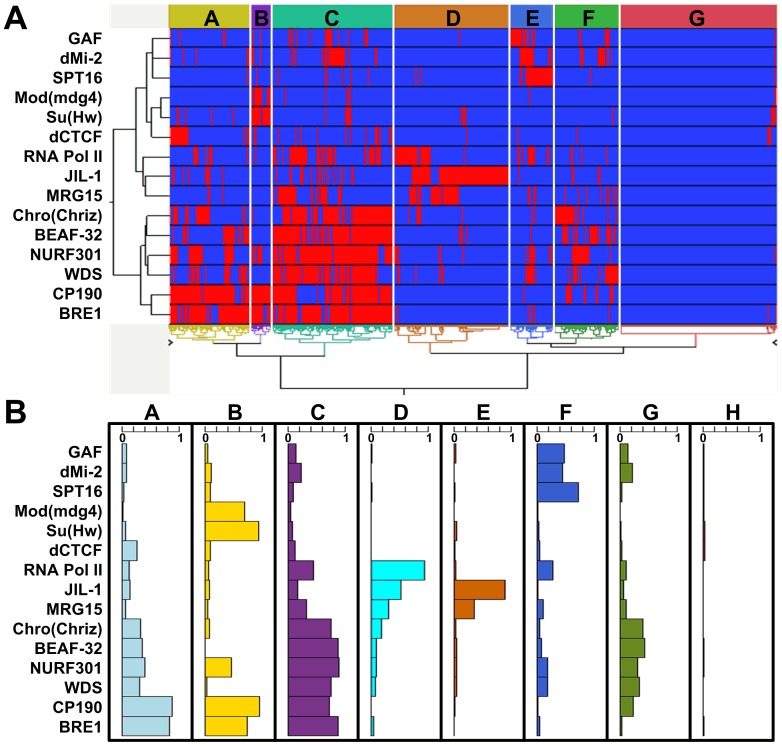
Subgroups of CTRs based on associated proteins in *Drosophila* S2 cell line. (A) Heat-map of the hierarchical clustering analysis result. Each column denotes a single CTR, and each row represents one protein included in the association analysis. The red and blue bars denote the presence or absence of an association with the corresponding CTR, respectively. Capital letters within colored boxes highlight the different subgroups of CTRs. (B) Proportions of CTRs in each subgroup (identified in (A)) that are associated with individual protein. The width of the bar indicates the percentage of CTRs in each group that are bound by the respective protein.

As mentioned above, the spatial relationship between CTR and the binding of known insulator proteins suggests that the CTRs observed for S2 cells are due to the barrier activity of insulator proteins. However, more than half of the CTRs are not co-localized with any of the known insulator proteins (Figure 2C). To gain a comprehensive understanding of the H3K27me3 CTRs identified in S2 cells, we expanded our analysis to include the binding profiles for other chromatin-associated proteins, all of which were generated by the modENCODE project [21] with the S2 cells. In this analysis we excluded proteins which have been shown to be directly involved in the establishment or maintenance of the facultative heterochromatin, such as the polycomb group proteins, the trithorax group proteins, and heterochromatin binding proteins. A total of 15 binding profiles were selected (Figure 3, Table S1 in File S1), and the binding call was processed as described [14]. Similar to previous association studies [27], we considered a binding within 1 kb of a CTR as a positive association.

We then conducted unsupervised hierarchical clustering to classify CTRs based on the association with these chromatin-associated proteins. From the clustering analysis, the predicted CTRs can be clearly divided into eight groups (Figure 3A). We also performed principal component analysis on the 15 proteins and the first three principal components turned out to account for 25.1%, 12.2% and 10.5% of the total variance respectively (Figure S4 in File S1). After projecting the predicted CTRs on the first three principal components, the eight distinct CTR groups were also clearly separated (Figure S4 in File S1), demonstrating that the grouping of CTRs was robust to different classification methods.

The protein occupancy in distinct CTR groups clearly suggested that the majority of CTRS in groups A, B and C are associated with the insulator protein CP190, whereas the other five groups are CP190 independent (Figure 3B). For the 3 CP190-associated groups, about 30% of CTRs in Group A are also associated with the insulator protein dCTCF, which requires CP190 as a co-factor [20]. The majority of CTRs in group B are also bound by insulator proteins Su(Hw), Mod(mdg4), which are the required trans factors for the *gypsy* insulator [18], [28], [29]. CTRs in Group C are enriched for insulator protein BEAF-32. Interestingly, CTRs in this group are also associated with the chromatin remodeling protein NURF, which has been shown to be required for establishing the chromatin barrier activity of cHS4 at the chicken β-globin locus [30]. Taken together, our unsupervised hierarchical clustering agrees very well with the model put forward based on genetic analysis of three insulator proteins, i.e. while the three insulator proteins Su(Hw), BEAF-32, and dCTCF barely overlap with each other, they all co-localize with CP190 [31].

Interestingly, the majority of CTRs in group E are associated with JIL1, which can maintain euchromatic state by terminating the constitutive heterochromatin spreading [32], [33]. The colocalization suggests that JIL1 may also antagonize facultative heterochromatin through a mechanism which is different from the other CTR groups. The separation of group D from group E is due to the presence of RNA polymerase II. However, CTRs in group D were not associated with annotated TSSs (Transcription Start Sites), while the majority of CTRs in groups A, C, and G are located close to TSS (Figure S5 in File S1). In depth analysis indicated that the Pol II binding “peaks” associated with CTRs in group D are much smaller than those associated with bona fide TSS and they do not correlate with H3K4me2/3 enrichment. Close inspection suggested that the association of Pol II binding with this group was questionable and could be due to artifact of peak calling. Since all of the peak calling were generated by modENCODE with unifying standard [14], we refrained from changing the calling specifically for the Pol II data. Group F is associated with the insulator protein GAF [34].

In groups G and H, most of the CTRs have no clear association with any of the investigated proteins, which suggests the existence of other proteins functioning at these CTRs. Interestingly, the novel chromatin barrier ILB we have recently identified [10] does not co-localize with any of the 15 proteins, and belongs to group H.

### Strong Co-factor Binding Distinguishes dCTCF and Su(Hw) Binding Associated with CTR vs. those in H3K27me3-enriched Regions

The strict spatial relationship between the binding of insulator proteins and the identified CTRs strongly suggests a cause-effect relationship between insulator protein binding and the formation of the boundary for the H3K27me3 modification. However, analysis of the global profiles indicated that only a small portion of binding sites for dCTCF and Su(Hw) are associated with CTRs. To reconcile the two seemingly conflicting observations, we first asked whether there is any difference in terms of binding intensity by the respective insulator proteins. To address this question, we compared the binding profiles at sites associated with CTRs against those in regions enriched for H3K27me3, which are clearly not associated with any chromatin barrier activity.

**Figure 4:**
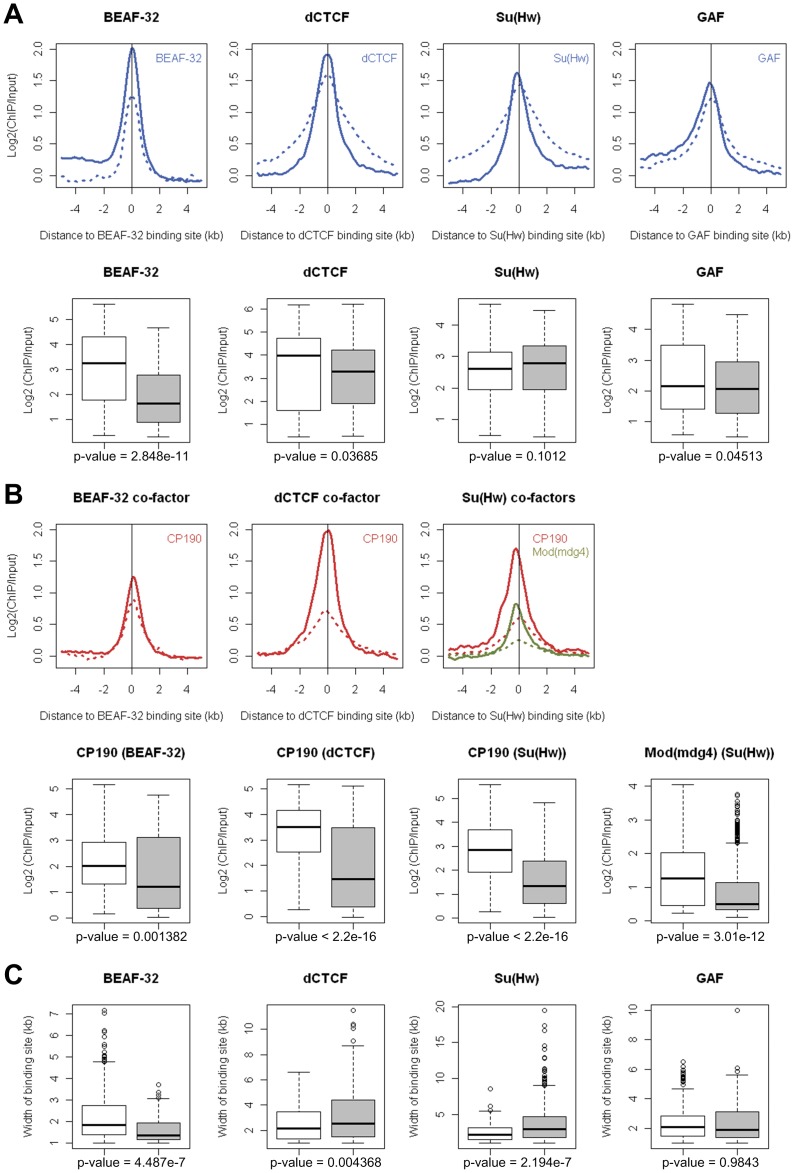
Binding intensity and patterns of insulator proteins and co-factors associated with CTRs in *Drosophila* S2 cell line. The enrichment levels of respective insulator proteins (A) and co-factors (B) around binding sites associated with CTR (solid lines) or located in H3K27me3-enriched region (dashed lines). For CTR-associated binding sites, negative and positive distances denote euchromatic and heterochromatic side. Box plots show the peak values for individual insulator proteins (A) and co-factors (B) at binding sites associated with CTR (open box) or in heterochromatic regions (shaded box). (C) Box plots of the width of insulator proteins binding patterns at binding sites associated with CTR (open box) or in heterochromatic regions (shaded box). P-values were all calculated by Wilcoxon rank sum test.

We found that for dCTCF, Su(Hw), and GAF, there was no significant difference in terms of enrichment levels at the peaks of the binding (Figure 4A, 2B). There was only marginal difference for BEAF-32, where the peak intensity was about 50% higher in sites associated with CTR (Figure 4A). These findings suggested that insulator proteins such as dCTCF and Su(Hw) can bind with similar affinity to euchromatic regions associated with CTRs and facultative heterochromatic regions enriched for H3K27me3. Although the intensities at the peak were similar for both dCTCF and Su(Hw), we did notice that the binding for these two insulator proteins was more spread in heterochromatic regions and more constrained in binding sites associated with CTRs (Figure 4A,C, 2B). The functional significance of this difference is unclear.

It has been well documented that the binding of co-factors such as CP190 is required for the enhancer blocking function of Su(Hw) and dCTCF [20], [29]. We found that the intensity of CP190 binding was much higher at sites associated with CTRs. This is true for both dCTCF and Su(Hw), where the CP190 binding intensities at sites associated with CTRs were significantly higher than those that are in H3k27me3-enriched regions (Figure 4B, 2B). Significant difference in binding intensity was also observed for another co-factor of Su(Hw), i.e. Mod(mdg4) [28], for which the binding intensity for sites associated with CTRs was 2.98 fold of those in heterochromatic regions (Figure 4B). These observations strongly suggested that co-factors such as CP190 and Mod(mdg4) are involved in establishing the chromatin barrier activity of dCTCF and Su(Hw).

### Poly(dA:dT) Tracts and Decreased Nucleosome Density Around the Insulator Binding Sites Associated with CTR

**Figure 5:**
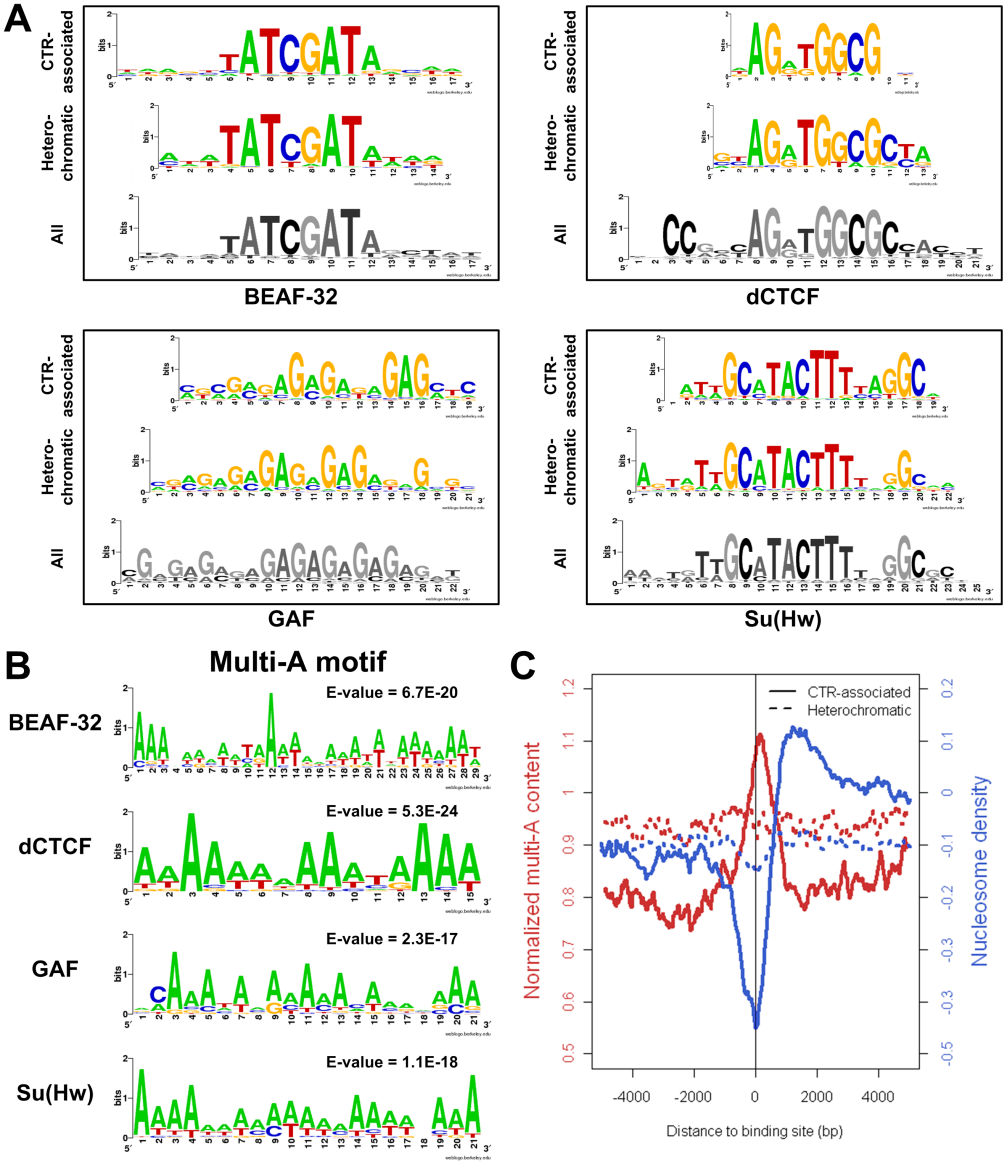
Cis-elements associated with CTRs in *Drosophila* S2 cell line. (A) Logos representation of motifs identified from DNA sequences underlying insulator protein binding sites associated with CTRs (CTR-associated) or in H3K27me3-enriched (Heterochromatic) regions. Motifs obtained with all binding sites are represented at the bottom. (B) Multi-A motifs are the discriminative motif identified by MEME for CTR-associated binding sites vs. heterochromatic binding sites. (C) Multi-A (AAAA/TTTT) content (normalized to genome average, red curve) and nucleosome density (blue curve) around CTR-associated insulator protein binding sites (solid line) and heterochromatic binding sites (dashed line). Data presents combined value for all the insulator proteins, dCTCF, Su(Hw), GAF, and BEAF-32. For CTR-associated binding sites, negative and positive distances denote euchromatic and heterochromatic side.

We next asked whether there is any difference between the DNA sequences underlying the insulator protein binding sites associate with CTRs and those in H3K27me3-enriched regions. We inputted the 400 bp regions around the CTR-associated binding sites to CisFinder [35] to identify statistically overrepresented DNA motifs, which were then compared with the motifs obtained with the 400 bp sequences surrounding the heterochromatic (H3K27me3-enriched) binding sites. There was no significant difference between the motifs identified from CTR-associated binding sites vs. those identified from the binding sites in heterochromatic region (Figure 5A). In fact, motifs identified from the aforementioned two subsets resembled the motifs identified using all binding sites identified in the S2 cells. This suggested that the DNA sequences interacting with the insulator proteins do not distinguish whether the association of the respective insulator protein can function as chromatin barrier or not.

We then asked whether sequences surrounding the CTR-associated insulator protein binding sites have discriminative patterns comparing to those surrounding the heterochromatic binding sites. Interestingly, when we supplied MEME [36] with the CTR-associated binding sites as positive regions and the heterochromatic binding sites as negative regions to identify discriminative motifs, a motif with continues deoxyadenosine (multi-A) showed up for all of the four insulator proteins (Figure 5B). Similar results were obtained when using the CisFinder program [35]. This indicated that a key distinction of insulator protein binding sites associated with CTRs was that they tend to be in close proximity to sequences with long stretch of dA/dTs (poly(dA:dT) tracts).

DNA sequences with poly(dA:dT) tracts where n(A/T)≥4 has been found to be rigid and discourage nucleosome binding [37], [38]. When we compiled the levels of poly(dA:dT) (frequency of AAAA/TTTT) and the nucleosome density around the binding sites of the four known insulator proteins (Su(Hw), BEAF-32, GAF, dCTCF), we found that the binding sites associated with CTRs were strongly associated with increased poly(dA:dT) levels as well as dramatically decreased nucleosome occupation (increased sensitivity to MNase) (Figure 5C). In contrast, such an association was not observed for binding sites in H3K27me3-enriched regions. We concluded that the CTR-associated insulator protein binding sites tend to be surrounded by DNA sequences characterized with nucleosome-destabilizing poly(dA:dT) tracts and manifest as hypersensitive to MNase.

### Poly(dA:dT) Tracts and Increased Sensitivity to MNase are Associated with CTRs that do not Bind with known Insulator Proteins

**Figure 6:**
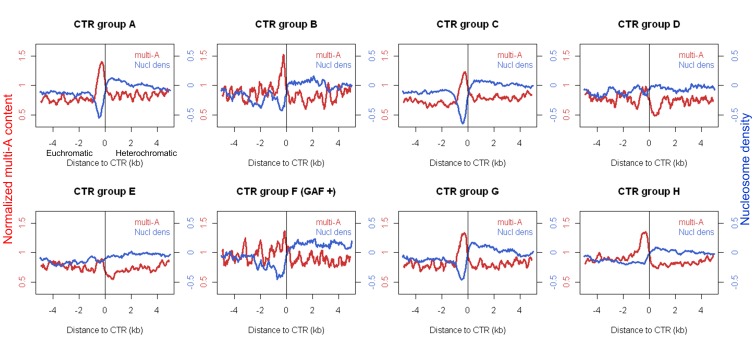
Multi-A (AAAA/TTTT) content (normalized to genome average, red curve) and nucleosome density (blue curve) around individual subgroup of CTRs in *Drosophila* S2 cell line (For group F only those co-localized with GAF were included). The negative and positive distances denote the euchromatic and heterochromatic sides of CTR, respectively.

To see how general are poly(dA:dT) tracts and increased MNase-sensitivity associated with CTRs of H3K27me3, we plotted the distribution for each of the groups identified by the hierarchical clustering (Figure 3A). We found that for CTRs in groups A, B, and C, which are all enriched for the binding of CP190, there is a clear trend of increased level of poly(dA:dT) (n≥4) and decreased nucleosome occupancy. The region of increased poly(dA:dT) levels roughly correlates with that of the increased sensitivity to MNase, and both peak at the euchromatic side of CTRs (Figure 6). CTRs in group A, B, and C are enriched for the presence of binding of dCTCF, Su(Hw), and BEAF-32, respectively. However, not all of the CTRs in each group are associated with the corresponding insulator protein (Figure 3B).

We could not observe clear increase of poly(dA:dT) level, nor decreased nucleosome density, associated with the CTRs in groups D and E, which are associated with PolII and JIL1, respectively. While there is a slightly increased level of poly(dA:dT) and a decreased level of nucleosome density for CTRs in group F. However, the distribution is somewhat different from those observed for groups A, B, and C, in that there is no clear peak.

Interestingly, for CTRs in groups G and H, which are not enriched for the binding of any known insulator proteins or other chromatin-associated proteins investigated here, there is a clear trend of increased poly(dA:dT) level on the euchromatic side of CTRs. For group G, there is also a significant decrease of nucleosome density correlates with the increased level of poly(dA:dT). It is well known that nucleosome positioning sequences, including poly(dA:dT), are associated with promoters [39]. However, the majority of CTRs in group H are not close to TSS (Figure S5 in File S1) or associated with Pol II binding, the increased multi-A level in the two groups is unlikely due to the nucleosome positioning sequences associated with promoters. This indicated that the presence of poly(dA:dT) tract and decreased nucleosome density is a general feature of CTRs beyond those that associate with the characterized insulator proteins.

### Enrichment of H3.3 but Decreased Nucleosome Turnover at CTR-associated dCTCF Binding Sites

It has been shown in mammalian systems that the binding of insulator proteins such as CTCF results in dynamic (unstable) nucleosomes and manifest as sites with increased enrichment of histone variants such as H3.3/H2A.Z at low salt isolation condition [40]. The dynamics of nucleosomes in S2 cells has also been assayed with the rate of histone variant H3.3 replacement [41], and more recently, with the CATCH-IT technology [42]. The latter is based on metabolic labeling of histones and is thus a direct measurement of nucleosome turnover rate independent of the composition of nucleosome. It has been shown that in general the profiles obtained with CATCH-IT correlate very well with the one based on H3.3 incorporation [42]. In addition to the CATCH-IT profile, datasets for H3.3 enrichment at low vs. high salt isolation conditions [43], nucleosome density (ratio of nucleosomal/genomic) [43], and DNA accessibility evaluated with methylation footprinting [44] were also available for the same cell line.

**Figure 7:**
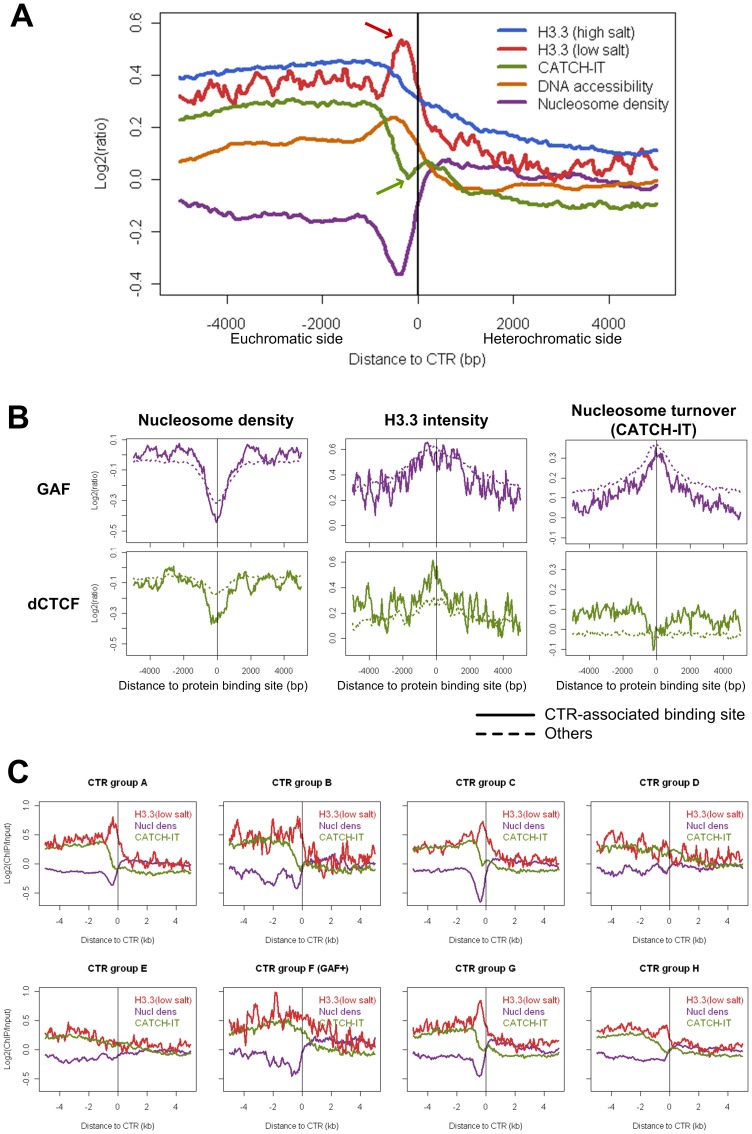
Contrasting patterns of H3.3 enrichment and nucleosome turnover rate associated with subgroups of CTRs in *Drosophila* S2 cell line. (A) Composite plot for all CTRs. H3.3 (low salt) incorporation is enriched on the euchromatic side of CTRs (red arrow), while nucleosome turnover rate (CATCH-IT) is drops down sharply at the same region (green arrow). (B) H3.3 enrichment and CATCH-IT measurements of nucleosome turnover rate moves to the same direction for GAF (both CTR-associated and others). In contrast, for CTR-associated dCTCF binding sites, the enrichment of H3.3 is accompanied by decreased turnover rate. (C) Plots of H3.3 enrichment (red), nucleosome turnover rate (green, measured with CATCH-IT), and nucleosome density (purple) for each subgroup of the CTRs (for group F only those co-localized with GAF were included). Note the contrasting pattern between H3.3 enrichment and CATCH-IT profile in subgroups A, B, C, G, but not in subgroups D and E.

When these profiles were evaluated around all of the H3K27me3 CTRs identified for S2 cells, we found that there was a conspicuous decrease of nucleosome density at the euchromatic side of CTRs (Figure 7A). The lowest point of nucleosome density is about 2 nucleosomes away from the CTR, which corresponds well with the peak of the binding sites for known insulator proteins (Figure 2D), as well as the region enriched for poly(dA:dT) tracts (Figure 6). Correspondingly, consistent increase of DNA accessibility was also observed at the same relative position.

However, when the nucleosome dynamics data was evaluated, we noticed an apparent discrepancy between the H3.3 incorporation measurements and the CATCH-IT profiles. At the same relative location to CTRs, there is a significant increase of H3.3 incorporation (at low salt condition), which would have indicated an increased dynamics (turnover rate) at the insulator protein binding sites. However, this was contradicted by the CATCH-IT profile at these sites, which showed a sharp drop at the same relative position (Figure 7A).

To understand the cause of this discrepancy between the H3.3 incorporation and the turnover rate measured with CATCH-IT, we looked at these profiles associated with each individual insulator proteins (Figure 7B). We found that for the GAF binding sites, whether associated with CTRs or not, there is a consistent increase of both H3.3 and CATCH-IT. This agrees well with previous findings that GAF binding sites are marked by increased nucleosome dynamics [42]. This also indicates that in terms of nucleosome dynamics, there is no difference between GAF binding sites associated with CTRs vs. those that are not associated.

However, a contrasting pattern was specifically observed between the H3.3 and CATCH-IT profiles around CTR-associated dCTCF binding sites. While there is a significant increase of H3.3 in these sites, the CATCH-IT data indicated that the turnover rate at these sites is not higher, but rather lower than the neighboring region (Figure 7B). This contrasting trend of H3.3 incorporation and nucleosome turnover rate suggested that, unlike GAF binding sites, the increased level of H3.3 incorporation is accompanied by decreased level of nucleosome turnover at the dCTCF binding sites close to CTRs. Interestingly, this contrasting trend was only obvious with dCTCF binding sites associated with CTRs, but was not observed around dCTCF binding sites not associated with a CTR (more than 1 kb away from the closest CTR) (Figure 7B).

As aforementioned, the contrasting pattern between the enrichment of H3.3 and decreased nucleosome turnover rate was obvious when the two profiles were evaluated for all H3K27me3 CTRs identified in S2 cells. For the groups of CTRs associated with known insulator factors, we found that this contrasting pattern is most prominent for group A (Figure 7C). About 30% of CTRs in this group has verified binding of dCTCF (Figure 3B). In addition, the enrichment of H3.3 was also prominent for CTRs in group G, which has no clear association with any of the known insulator proteins.

### Chromatin Transitional Regions in the HeLa Cell Line

**Figure 8:**
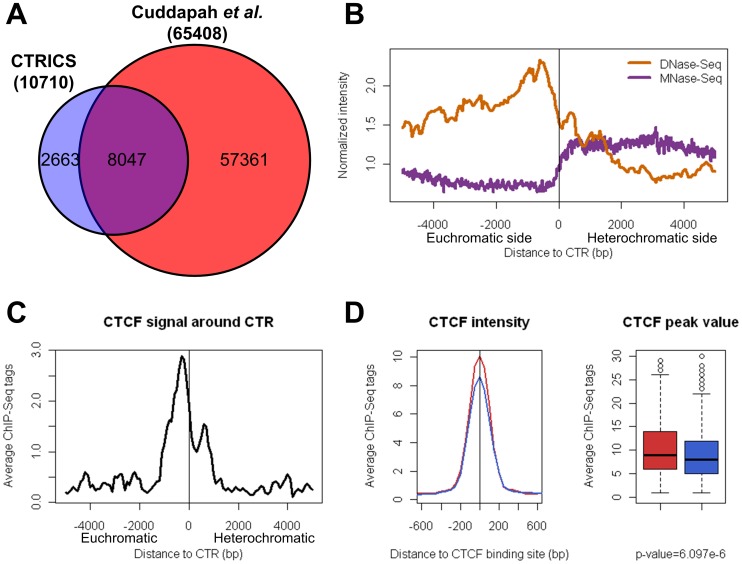
Chromatin transitional regions in human HeLa cell line. (A) 6852 predicted CTRs in HeLa cells are overlapping (within 1 kb) with the chromatin barrier regions in the previous study. (B) DNA accessibility (measured by DNase-Seq) and nucleosome density (measured by MNase-Seq) around all the predicted CTRs in HeLa cell line (normalized to genome average). The negative and positive distances denote the euchromatic and heterochromatic sides of CTR, respectively. (C) Binding pattern of insulator protein CTCF around the CTRs which co-localize with CTCF. The negative and positive distances also denote the euchromatic and heterochromatic sides of CTR, respectively. (D) The enrichment level of CTCF around CTR-associated (red) and heterochromatic (blue) binding sites. Box-plots show the peak values of CTCF at the CTR-associated (red) and heterochromatic (blue) binding sites. The peak values of CTCF at CTR-associated binding sites were significantly greater than that at the heterochromatic binding sites (p-value = 6.097e-6, Wilcoxon rank sum test).

Applying the CTRICS program to H3K27me3 ChIP-Seq dataset derived from human HeLa cells [27] identified a total of 10710 CTRs. The majority (8047) of which overlaps with the boundaries of H3K27me3 domains identified by Cuddapah *et al.* [27], which identified a total of 32,704 H3k27me3 domains in HeLa cells (Figure 8A). The difference in the number of H3k27me3 boundaries identified by CTRICS and the H3K27me3 domain approach is likely due to the combined effect of 1.) the CTRICS methodology is less sensitive to fluctuation of H3K27me3 enrichment levels within H3k27me3-enriched domains (Figure 1C); and 2.) CTRICS is more stringent in that it will only identify boundaries with a significant drop of H3K27me3 level (Figure S6 in File S1).

With this stringent set of CTRs in HeLa cells, there is a significant increase of DNA accessibility (DNase-Seq data set from [45]) in at the immediate euchromatic side of the CTRs (Figure 8B). This is very similar to what we observed in the S2 cells. There is also a significant change of nucleosome density (MNase-Seq data from [46]) around the predicted CTRs, which confirms that our method is identifying well defined facultative heterochromatin boundaries.

Similar to what was observed for S2 cells, CTCF was also enriched on the euchromatic side of CTRs with about 2 nucleosomes in between (Figure 8C). However, unlike dCTCF, there was a minor peak of the polled CTCF binding signal on the heterochromatic side of CTRs (Figure 8C). Interestingly, a similar major peak and minor peak pattern of CTCF binding was also observed independently for facultative heterochromatin boundaries in human CD4^+^ cells identified with a consortium of histone modification profiles and a maximal segment algorithm [26]. Overall the binding intensity for CTCF was moderately, but significantly, higher for binding sties associated with CTR than those in heterochromatic regions (Figure 8D). Since no co-factor such as CP190 was identified in mammalian systems, which prevented us to test whether similar distinction of co-factor binding also applies to human CTRs.

## Discussion

In this work, we showed that it was possible to identify the boundaries of facultative heterochromatin based on H3K27me3 ChIP-Seq data. Our two-tiered method first identifies a heterochromatin to euchromatin transition event by considering the enrichment value for a relatively large region. Following that, the 200 bp region that shows the greatest transition rate of enrichment values is designated as the CTR. The validity of this simple strategy was firstly verified by the dramatic difference in active/repressive histone modifications and gene expression levels on the heterochromatic vs. euchromatic side of the predicted CTRs (Figure 1). More importantly, the validity of this strategy was vindicated by the fact that, for CTRs associated with the binding of known insulator proteins, there is a strict spatial relationship between the CTRs and the insulator protein binding sites.

### Fixed vs. Variable Boundary for Facultative Heterochromatin

The method developed in this study is specifically suitable for the identification of fixed boundaries for facultative heterochromatin. Visual inspection of H3K27me3 profile has suggested that certain H3K27me3 domains do not have a fixed boundary [47]. It is clear that for constitutive heterochromatin close to centromere, the boundary of heterochromatin marked by H3K9me2/3 can vary in different cells of the same tissue. This phenomenon was reflected as “variegated” expression of reporter/marker genes located close to centromere, i.e. position-effect variegation (PEV) (reviewed in [48], [49]). It is possible that our method won’t be sufficient to identify boundaries that show variable locations in individual cells, for which the pooled ChIP-Seq data will lack a sharp transition region.

It is conceivable that due to its close association with euchromatic region, the boundaries of facultative heterochromatin need to be precisely defined to avoid the disruption of the transcriptional regulation of adjacent genes. In the case of the cHS4 chromatin barrier in the chicken β-globin locus, the binding of USF1 was responsible for recruiting histone modifying enzymes which in turn catalyze euchromatic histone modifications on adjacent nucleosomes [8], [9]. The USF1-directed euchromatic histone modifications effectively block the propagation of heterochromatic marks and results in a sharp transition of histone marks. Interestingly, a recent study revealed that NURF is recruited by USF1 to cHS4 and is required for establishing the chromatin barrier [30]. Our analysis indicated that the binding of NURF (NURF301, Figure 3) is associated with CTRs in groups A, B, C, and G. It has been shown that *Drosophila* NURF is required for the enhancer-blocking activity of several insulators [50]. Our results suggest that its role in establishing chromatin barrier is also likely conserved over long evolutionary distance.

Our analysis of genome-wide H3K27me3 CTRs in S2 cells indicated that at least in this cell line, many boundaries of facultative heterochromatin, marked by the transition of H3K27me3 enrichment level, can be clearly identified. However, formation of facultative heterochromatin is, by definition, cell type specific. We found that clear boundaries cannot be reliably identified from H3K27me3 data obtained from homogenized animals (embryos or larvae). Since the binding profile of many insulator proteins as well as other epigenomic profile has been well studied in the S2 cells, the genome-wide identification of CTRs in this cell line allowed us to address several interesting questions in regards to chromatin barriers.

### Binding of Insulator Protein alone is not Sufficient for Establishing the H3K27me3 Boundary

Our analysis indicated that only a small portion of genome-wide binding sites for insulator proteins such as dCTCF and Su(Hw) are associated with the CTRs. This was not surprising, given that a genomic study conducted in mammalian cells also revealed that for CTCF binding sites observed for CD4+ T cells and HeLa cells, only a small percent (about 5.6% and 4.1%, respectively) are associated with the boundaries of H3K27me3-enriched domains [27]. Our results indicated that similar to what was observed for CTCF in mammalian cells, the majority binding sites for insulator proteins such as dCTCF and Su(Hw) do not co-reside with the boundaries of facultative heterochromatin. The same mammalian study also revealed that only a very small portion (less than 5%) of the H3K27me3 boundaries in those cells have a CTCF binding site within 1 kb of distance. Although many more insulator proteins have been characterized in *Drosophila*, less than half of all H3K27me3 CTRs identified in S2 cells are associated with any of the known insulator proteins. This indicates that uncharacterized mechanisms, which do not involve any of those proteins known to play a role in this process, is responsible for establishing more than half of the facultative heterochromatin boundaries in S2 cells.

In this study, by narrowing down the transitional region to 200 bp, we were able to reveal some very interesting relationships between the binding of insulator proteins and the CTRs. Central to these findings are the observation that there is a clear spatial relationship between the binding sites of insulator proteins and the CTRs. The binding of insulator proteins is at the euchromatic side of CTRs and the peak of binding is about 1–2 nucleosomes away from the CTRs. This strict spatial relationship suggests that there is a functional relationship between the binding of these insulator proteins and the establishment of the sharp transition at the CTRs.

A prominent question in regards to insulator proteins binding and the formation of chromatin boundary is what distinguishes those sites associated with a chromatin boundary versus those do not. We found that compared with dCTCF and Su(Hw) binding sites in heterochromatic regions, the binding sites associated with CTRs were bound by higher levels of co-factors such as CP190 or/and Mod(mdg4). In contrast, such a distinction was not observed for CTRs associated with BEAF-32. A recent work revealed that, unlike dCTCF and Su(Hw), binding of CP190 at BEAF-32 binding sites was not affected when the insulator protein was knocked down [47]. The same work also suggested that the inherited binding preferences of, not the interaction between, the two proteins could be responsible for the observed colocalization of BEAF-32 and CP190. Our observations further support their argument. The increased binding intensities of co-factors at dCTCF and Su(Hw) sites associated with CTRs were not simply because those binding sites are located on the euchromatic side of the CTRs, since the intensity at CTR-associated sites was significantly higher when compared with that in euchromatic regions (Figure S7 in File S1). These observations strongly suggested that there is a significant difference in co-factor binding between CTR-associated binding of dCTCF and Su(Hw) vs. those that are in heterochromatic regions.

Besides the difference in co-factor binding, the underlying DNA sequences surrounding CTR-associated binding sites are enriched for poly(dA:dT) tracts. Poly(dA:dT) tracts have been found to form rigid structures and discourage nucleosome formation [37], [39]. The fact that poly(dA:dT) tracts distinguish CTR-associated insulator protein binding sites from those in heterochromatic region suggested that it plays a role in establishing/encouraging the barrier function of dCTCF and Su(Hw). One hypothetic model come out of our analysis is that the presence of nucleosome-destabilizing sequences flanking the insulator protein binding site associated with CTRs could change the dynamics of nucleosome formation as well as facilitate increased binding of co-factors. However, the enrichment of poly(dA:dT) tracts surrounding CTR-associated binding sites could simply be an indicator of nucleosome depletion, instead of playing a role in the formation of nucleosome depletion regions, as suggested by a recent study that these regions favor G/C to A/T mutations [51].

### Nucleosome Dynamics, Histone Variants, and H3K27me3 Boundary

Increased nucleosome dynamics, often manifested as increased enrichment level of histone variants such as H3.3, has been linked with transcriptionally active genes in both *Drosophila* and mammalian systems. Our analyses indicated that distinctive patterns of nucleosome dynamics and histone variants incorporation are associated with different subgroups of CTRs.

**Figure 9:**
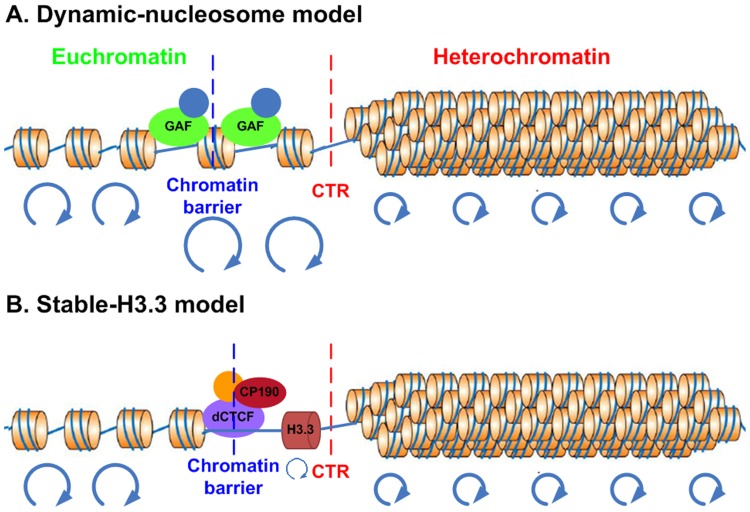
Proposed models for facultative heterochromatin boundary. Models represent distinct features of GAF-associated (A) vs. dCTCF-associated (B) CTRs. The red and blue dashed lines denote the position of CTR and chromatin barrier, respectively. The blue circles at the bottom of each model indicate the nucleosome turnover rate, the bigger the circles, the faster the nucleosomes turnover. For dCTCF-associated CTRs, the increased enrichment of H3.3 is coupled with decreased turnover rate.

For CTRs associated with GAF, there is an increased nucleosome turnover rate (measured by CATCH-IT) as well as an enrichment of H3.3 incorporation (Figure 7B). This agreement between turnover rate measured by CATCH-IT and H3.3 incorporation has been observed globally for TSSs (transcription start sites) and several important chromatin landmarks such as binding sites for ploycomb group proteins [42]. It is conceivable that the dynamic nucleosome located at the binding site of GAF could serve to discourage the propagation of repressive histone modifications (Figure 9A), which is in consistent with the model proposed in yeast [52].

However, a surprising phenomenon was identified for those CTRs that are associated with dCTCF. Instead of increased turnover rate, the nucleosomes close to the binding sites actually showed decreased level of turnover as measured by CATCH-IT (Figure 7B). This reduced turnover rate at H3K27me3 CTRs was not limited to those that have binding of dCTCF. It was also prominent for CTRs in group G (Figure 7C). Intriguingly, the decreased level of turnover is accompanied by increased incorporation of H3.3 in those CTRs. This suggested that for certain subgroups of CTRs, the nucleosome at the boundary has reduced turnover rate but nonetheless has strong preference for the histone variant H3.3 (Figure 9B). The preference of H3.3 could potential serve as a deterrent for the spreading of H3K27me3. However, this mechanism, if indeed contributes to the formation of H3K27me3 boundary, is likely redundant and dispensable. Since the deletion of H3.3 did not have significant impact on facultative heterochromatin formation and can be compensated by overexpression of H3 [53].

## Materials and Methods

### CTRICS (Chromatin Transitional Regions Inference from ChIP-Seq) Algorithm

#### ChIP-Seq data preprocessing

The program will take H3K27me3 ChIP-Seq data as input (and control dataset measures the input DNA level, if available). The datasets should be in BED (Browser Extensible Data) format. Redundant tags which map to the same genomic region will be kept as a single tag in order to minimize potential PCR bias. CTRICS then divides the whole genome into non-overlapping windows of size *w* (default is 200 bp), counts ChIP-Seq tags in each window and generates a bedGRAPH file, which can be viewed with the UCSC Genome Browser [54].

#### Scoring system

We measure the rate of chromatin transition with T-score as,
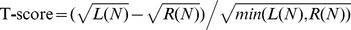
where *L(N)* and *R(N)* denote the average of ChIP-Seq tags (when there is no input control file), or normalized ChIP-Seq tags in the *N* (default = 20) windows upstream and downstream of the given window, respectively. Because H3K27me3 generally forms broad regions covering repressive genes and intergenic regions [55], [56], the long genomic region used in this initial evaluation should minimize the impact of enrichment level fluctuation observed for H3K27me3 enriched regions. Similar to other studies analyzing the change of chromatin modifications [57], [58], we took the square root transformation to minimize the variance introduced by higher counts. We will take the denominator as 1/*N* if the min(L(N), R(N)) was zero. A T-score greater than the threshold (see below), and has one side show significant enrichment of ChIP-Seq tags, will be taken as a candidate region where a transition exist.

In the next step, in seeking to pinpoint the CTR location, we calculate the T’-score for each window around the candidate CTRs, and the region that has the highest T’-score, i.e. the highest enrichment transition rate, will be reported as the predicted CTRs. T’-score is defined as,
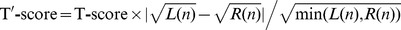
which is the product of the T-score for the long genome region (*N* windows) and the absolute T-score for a short genome region (*n* windows; default = 3).

#### Estimation of T-score threshold without control file

To assess the statistical significance of each CTR (the probability that the observed T-score is by chance), we need to derive the distribution of T-score in the background model. In this program, we chose not to make any assumption about the background distribution of the ChIP-Seq tags because different datasets have variations and will not always follow a certain assumed distribution. Instead we applied a bootstrapping approach to get the background distribution of T-score. The bootstrapping was conducted by randomly choosing (with replacement) *N* windows from the whole genome as left windows and *N* windows as right windows, then calculating T-score with the randomly chosen windows. The T-score distribution in the random background model is obtained by repeating (with replacement) the above process for a large number of times (10^6^). Based on the T-score distribution and the runtime input p-value, we will get the T-score threshold.

#### Estimation of T-score threshold with control file

In the presence of the input control file, an extra step is needed before the estimation of T-score threshold and prediction of CTRs. We named this step as “background correction”, which normalizes the tag number of each window in ChIP file to the tag number of the same window in control file by the following formula,
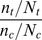
where *n_t_*, *n_c_* are the tag numbers of a given window in ChIP file and control file (again *n_c_* will be set as one if it is zero), *N_t_*, *N_c_* are the total tag counts in ChIP and control files. After the background correction, the program will use the normalized tag counts to estimate T-score cutoff and to predict CTRs. Figure S8 in File S1 shows the workflow of CTRICS.

CTRICS has been implemented in Perl, and it can be downloaded from the following link http://159.178.28.30/CTRICS/home.htm.

### Dataset

The datasets used in this study are listed in Table S1 and S2 in File S1.

### Parameters used for Predicting CTRs and/or H3K27me3 Domains

CTRs were predicted in *Drosophila* S2 and human HeLa cells by CTRICS with default parameters (expect that the p-value was set to 0.005 for HeLa cells). We ran SICER without control file using default parameters suggested by the authors (window size = 200 bp, gap size = 600 bp, E-value = 100, p-value = 0.2), and we took the effective *Drosophila* genome size as 71.6%. RSEG was also run with default parameters defined by the program. The two boundaries of an H3K27me3 enrichment domain predicted by these programs were taken as two CTRs, and we discarded the boundaries which are less than 4 kb to an unmappable region.

### Statistical Analysis

The two-way hierarchical clustering (Ward method) and principal component analysis were carried out using JMP Genomics 5.0 (SAS Institute, Cary, NC). A binding is considered positively associated with a CTR if the midpoint of the binding is within 1 kb of the CTR.

Wilcoxon rank sum test was performed in R programming environment (R version 2.9.2, R Development Core Team, 2009) to compare the gene expression levels on different sides of CTRs, as well as the binding intensity and width of binding sites of insulator proteins and co-factors.

### Motif Discovery

The insulator protein binding motifs were identified using CisFinder [35] with default setting. 400 bp regions centered on the midpoint of the bindings were used as input. The predicted motifs were depicted as color logos using WebLogo [59]. The discriminative motifs were discovered using MEME (web-version 4.8.1) [36].

### Calculation of Nucleotides Content

Poly(dA:dT) (AAAA/TTTT) level was calculated using a sliding window approach with window size of 200 bp and step of 25 bp. The contents were further normalized to the genome average.

## Supporting Information

File S1 **Figures S1–S8 and Tables S1–S2. Figure S1.** Construction of empirical positive and negative evaluation datasets. For the optimization of the parameters, we set up the empirical positive and negative evaluation datasets for CTR. For the positive dataset, we took the genomic loci which, (1) show clear H3K27me3 enrichment transition (H3K27me3 tags in the 4 kb region on the enriched and depleted side should be greater than 1.5 fold or less than 25% of genome average, respectively), (2) has a single enrichment region for one of the three known insulator proteins Su(Hw), dCTCF, and BEAF-32 and respective co-factors (CP190 and Mod(mdg4) for Su(Hw), and CP190 for dCTCF and BEAF-32) in the vicinity of the transition. With these criteria, we got 81 positive loci, among which 66, 8 and 7 are bound by BEAF-32, dCTCF and Su(Hw) respectively. For CTR-negative dataset, we used the following criteria, (1) a gene with corresponding genomic region greater than 5 kb and does not overlap with any other gene(s), and (2) the gene should have no alternative splicing, and (3) the average H3K27me3 ChIP-Seq tags in the transcribed region of the gene should be more than two-fold of the whole genome average. We scanned all the annotated genes, 159 of them went through this unbiased selection for S2 cells. To avoid possible chromatin barriers at TSS (transcription start site) or TES (transcription end site), we took the central 60% regions of each gene to form the negative dataset. Sample regions in positive and negative evaluation datasets. (A) A sample positive region on chromosome 3R. The region has enrichment of DNA-binding insulator protein Su(Hw) and co-factors CP190 and Mod(mdg4), while it does not have enrichment of other two DNA-binding insulator proteins (dCTCF, BEAF-32). In addition, there is dramatic transition in terms of H3K27me3 signal around this region. The dashed line indicates the midpoint of Su(Hw) enriched region, and it was also taken as the coordinate of this positive region. (B) A sample negative region on chromosome 2L. The entire transcribed region of the gene *CG4341* is covered by high level of H3K27me3. The central 60% of this gene (the region between two dashed lines) was taken as a negative region. **Figure S2.** Comparison of CTRICS with SICER and RSEG. (A) The Venn diagram shows the number of CTRs (predicted by CTRICS) that are overlapping with the chromatin boundaries predicted by the other two methods in S2 cells. (B) False-positive and false-negative rates for different methods based on the empirical evaluation datasets. (C) A region shows several examples of CTRs predicted by CTRICS (red bar), H3K27me3 boundaries predicted by RSEG (grey bar), and H3K27me3 domains predicted by SICER. RSEG missed the three boundaries shown in the blue dashed block, which can be corroborated with RNA-Seq and H3K4me3 ChIP-chip data. **Figure S3.** Sequencing depth analysis. In order to test if the H3K27me3 ChIP-Seq dataset has reached saturation status and if sequencing depth has any influence on the CTR prediction, we conducted the sequencing depth analysis. We first randomly extracted a series of subsamples (10%, 20%, 30%, and so on until 90% of the original tags) from the H3K27me3 ChIP-Seq dataset without replacement. We then identified chromatin transitional regions in each subsample using CTRICS with default parameters. The x-axis of the plot represents the percentage of subsample tags compared to the total tags (∼2.8 x10^6^), and y-axis indicates the number of CTRs identified. **Figure S4.** Principal component analysis of CTRs based on association with the 15 proteins. (A) Percentage of total variance accounted for by individual principal components. (B) Two-dimensional projections onto the first three principal components. Different colors of the dots represent different groups of CTRs corresponding to the groups shown in the hierarchical clustering result (Figure 3A). **Figure S5.** Genomic distribution of CTRs. The average intensities of RNA polymerase II (A) and H3K4me3 (B) around individual groups of CTR. (C) The distribution of CTRs in each group. **Figure S6.** An example of 2 CTRs (red bars) predicted by CTRICS in human HeLa cells. The panel below CTR shows H3K27me3 domains predicted in Cuddapah *et al.* 2009. CTRICS identifies the boundaries with a significant drop of H3K27me3 level, but ignores the boundaries with minor changes in H3K27me3 signal. **Figure S7.** Binding patterns of co-factors are different for CTR-associated and euchromatic binding sites. Binding patterns of insulator proteins (A) and their co-factors (B) around CTR-associated (solid curve), heterochromatic (dotted curve) and euchromatic (break curve) binding sites in *Drosophila* S2 cells. For CTR-associated binding sites, negative and positive distances denote euchromatic and heterochromatic side. **Figure S8.** Flowchart of CTRICS. The green characters are the parameters needed in each step. **Table S1.** The list of ChIP-Chip profiles used in the clustering analysis. **Table S2.** The list of datasets used in this study.
(DOC)

## References

[1] Muller J, Verrijzer P (2009) Biochemical mechanisms of gene regulation by polycomb group protein complexes. Curr Opin Genet Dev 19: 150–158. 19345089 10.1016/j.gde.2009.03.001

[2] Schwartz YB, Pirrotta V (2007) Polycomb silencing mechanisms and the management of genomic programmes. Nat Rev Genet 8: 9–22. 17173055 10.1038/nrg1981

[3] Raab JR, Chiu J, Zhu J, Katzman S, Kurukuti S, et al (2012) Human tRNA genes function as chromatin insulators. EMBO J 31: 330–350. 22085927 10.1038/emboj.2011.406 PMC3261562

[4] Gaszner M, Felsenfeld G (2006) Insulators: exploiting transcriptional and epigenetic mechanisms. Nat Rev Genet 7: 703–713. 16909129 10.1038/nrg1925

[5] Geyer PK, Spana C, Corces VG (1986) On the molecular mechanism of gypsy-induced mutations at the yellow locus of Drosophila melanogaster. EMBO J 5: 2657–2662. 3096713 10.1002/j.1460-2075.1986.tb04548.x PMC1167166

[6] Roseman RR, Pirrotta V, Geyer PK (1993) The su(Hw) protein insulates expression of the Drosophila melanogaster white gene from chromosomal position-effects. Embo J 12: 435–442. 8382607 10.1002/j.1460-2075.1993.tb05675.x PMC413226

[7] Kahn TG, Schwartz YB, Dellino GI, Pirrotta V (2006) Polycomb complexes and the propagation of the methylation mark at the Drosophila ubx gene. J Biol Chem 281: 29064–29075. 16887811 10.1074/jbc.M605430200

[8] Huang S, Li X, Yusufzai TM, Qiu Y, Felsenfeld G (2007) USF1 recruits histone modification complexes and is critical for maintenance of a chromatin barrier. Mol Cell Biol 27: 7991–8002. 17846119 10.1128/MCB.01326-07 PMC2169148

[9] West AG, Huang S, Gaszner M, Litt MD, Felsenfeld G (2004) Recruitment of histone modifications by USF proteins at a vertebrate barrier element. Mol Cell 16: 453–463. 15525517 10.1016/j.molcel.2004.10.005

[10] Lin N, Li X, Cui K, Chepelev I, Tie F, et al.. (2011) A Barrier-only Boundary Element Delimits the Formation of Facultative Heterochromatin in Drosophila and Vertebrates. Mol Cell Biol. 10.1128/MCB.05165-11 PMC3133385 21518956

[11] Zhang Y, Lin N, Carroll PM, Chan G, Guan B, et al (2008) Epigenetic blocking of an enhancer region controls irradiation-induced proapoptotic gene expression in Drosophila embryos. Dev Cell 14: 481–493. 18410726 10.1016/j.devcel.2008.01.018 PMC2901920

[12] Gurudatta BV, Corces VG (2009) Chromatin insulators: lessons from the fly. Brief Funct Genomic Proteomic 8: 276–282. 19752045 10.1093/bfgp/elp032 PMC2742804

[13] Hon G, Ren B, Wang W (2008) ChromaSig: a probabilistic approach to finding common chromatin signatures in the human genome. PLoS Comput Biol 4: e1000201. 18927605 10.1371/journal.pcbi.1000201 PMC2556089

[14] Kharchenko PV, Alekseyenko AA, Schwartz YB, Minoda A, Riddle NC, et al (2011) Comprehensive analysis of the chromatin landscape in Drosophila melanogaster. Nature 471: 480–485. 21179089 10.1038/nature09725 PMC3109908

[15] Filion GJ, van Bemmel JG, Braunschweig U, Talhout W, Kind J, et al (2010) Systematic protein location mapping reveals five principal chromatin types in Drosophila cells. Cell 143: 212–224. 20888037 10.1016/j.cell.2010.09.009 PMC3119929

[16] Zang C, Schones DE, Zeng C, Cui K, Zhao K, et al (2009) A clustering approach for identification of enriched domains from histone modification ChIP-Seq data. Bioinformatics 25: 1952–1958. 19505939 10.1093/bioinformatics/btp340 PMC2732366

[17] Song Q, Smith AD (2011) Identifying dispersed epigenomic domains from ChIP-Seq data. Bioinformatics 27: 870–871. 21325299 10.1093/bioinformatics/btr030 PMC3051331

[18] Harrison DA, Gdula DA, Coyne RS, Corces VG (1993) A leucine zipper domain of the suppressor of Hairy-wing protein mediates its repressive effect on enhancer function. Genes Dev 7: 1966–1978. 7916729 10.1101/gad.7.10.1966

[19] Gilbert MK, Tan YY, Hart CM (2006) The Drosophila boundary element-associated factors BEAF-32A and BEAF-32B affect chromatin structure. Genetics 173: 1365–1375. 16648647 10.1534/genetics.106.056002 PMC1526658

[20] Mohan M, Bartkuhn M, Herold M, Philippen A, Heinl N, et al (2007) The Drosophila insulator proteins CTCF and CP190 link enhancer blocking to body patterning. EMBO J 26: 4203–4214. 17805343 10.1038/sj.emboj.7601851 PMC2230845

[21] Roy S, Ernst J, Kharchenko PV, Kheradpour P, Negre N, et al (2010) Identification of functional elements and regulatory circuits by Drosophila modENCODE. Science 330: 1787–1797. 21177974 10.1126/science.1198374 PMC3192495

[22] Gan Q, Schones DE, Ho Eun S, Wei G, Cui K, et al (2010) Monovalent and unpoised status of most genes in undifferentiated cell-enriched Drosophila testis. Genome Biol 11: R42. 20398323 10.1186/gb-2010-11-4-r42 PMC2884545

[23] Schones DE, Zhao K (2008) Genome-wide approaches to studying chromatin modifications. Nat Rev Genet 9: 179–191. 18250624 10.1038/nrg2270 PMC10882563

[24] Bilodeau S, Kagey MH, Frampton GM, Rahl PB, Young RA (2009) SetDB1 contributes to repression of genes encoding developmental regulators and maintenance of ES cell state. Genes Dev 23: 2484–2489. 19884255 10.1101/gad.1837309 PMC2779743

[25] Hon G, Wang W, Ren B (2009) Discovery and annotation of functional chromatin signatures in the human genome. PLoS Comput Biol 5: e1000566. 19918365 10.1371/journal.pcbi.1000566 PMC2775352

[26] Wang J, Lunyak VV, Jordan IK (2012) Genome-wide prediction and analysis of human chromatin boundary elements. Nucleic Acids Res 40: 511–529. 21930510 10.1093/nar/gkr750 PMC3258141

[27] Cuddapah S, Jothi R, Schones DE, Roh TY, Cui K, et al (2009) Global analysis of the insulator binding protein CTCF in chromatin barrier regions reveals demarcation of active and repressive domains. Genome Res 19: 24–32. 19056695 10.1101/gr.082800.108 PMC2612964

[28] Ghosh D, Gerasimova TI, Corces VG (2001) Interactions between the Su(Hw) and Mod(mdg4) proteins required for gypsy insulator function. EMBO J 20: 2518–2527. 11350941 10.1093/emboj/20.10.2518 PMC125459

[29] Pai CY, Lei EP, Ghosh D, Corces VG (2004) The centrosomal protein CP190 is a component of the gypsy chromatin insulator. Mol Cell 16: 737–748. 15574329 10.1016/j.molcel.2004.11.004

[30] Li X, Wang S, Li Y, Deng C, Steiner LA, et al (2011) Chromatin boundaries require functional collaboration between the hSET1 and NURF complexes. Blood 118: 1386–1394. 21653943 10.1182/blood-2010-11-319111 PMC3152501

[31] Bushey AM, Ramos E, Corces VG (2009) Three subclasses of a Drosophila insulator show distinct and cell type-specific genomic distributions. Genes Dev 23: 1338–1350. 19443682 10.1101/gad.1798209 PMC2701583

[32] Bao X, Deng H, Johansen J, Girton J, Johansen KM (2007) Loss-of-function alleles of the JIL-1 histone H3S10 kinase enhance position-effect variegation at pericentric sites in Drosophila heterochromatin. Genetics 176: 1355–1358. 17435241 10.1534/genetics.107.073676 PMC1894597

[33] Zhang W, Deng H, Bao X, Lerach S, Girton J, et al (2006) The JIL-1 histone H3S10 kinase regulates dimethyl H3K9 modifications and heterochromatic spreading in Drosophila. Development 133: 229–235. 16339185 10.1242/dev.02199

[34] Schweinsberg S, Hagstrom K, Gohl D, Schedl P, Kumar RP, et al (2004) The enhancer-blocking activity of the Fab-7 boundary from the Drosophila bithorax complex requires GAGA-factor-binding sites. Genetics 168: 1371–1384. 15579691 10.1534/genetics.104.029561 PMC1448804

[35] Sharov AA, Ko MS (2009) Exhaustive search for over-represented DNA sequence motifs with CisFinder. DNA Res 16: 261–273. 19740934 10.1093/dnares/dsp014 PMC2762409

[36] Bailey TL, Boden M, Whitington T, Machanick P (2010) The value of position-specific priors in motif discovery using MEME. BMC Bioinformatics 11: 179. 20380693 10.1186/1471-2105-11-179 PMC2868008

[37] Suter B, Schnappauf G, Thoma F (2000) Poly(dA.dT) sequences exist as rigid DNA structures in nucleosome-free yeast promoters in vivo. Nucleic Acids Res 28: 4083–4089. 11058103 10.1093/nar/28.21.4083 PMC113125

[38] Mavrich TN, Ioshikhes IP, Venters BJ, Jiang C, Tomsho LP, et al (2008) A barrier nucleosome model for statistical positioning of nucleosomes throughout the yeast genome. Genome Res 18: 1073–1083. 18550805 10.1101/gr.078261.108 PMC2493396

[39] Mavrich TN, Jiang C, Ioshikhes IP, Li X, Venters BJ, et al (2008) Nucleosome organization in the Drosophila genome. Nature 453: 358–362. 18408708 10.1038/nature06929 PMC2735122

[40] Jin C, Zang C, Wei G, Cui K, Peng W, et al (2009) H3.3/H2A.Z double variant-containing nucleosomes mark 'nucleosome-free regions' of active promoters and other regulatory regions. Nat Genet 41: 941–945. 19633671 10.1038/ng.409 PMC3125718

[41] Mito Y, Henikoff JG, Henikoff S (2005) Genome-scale profiling of histone H3.3 replacement patterns. Nat Genet 37: 1090–1097. 16155569 10.1038/ng1637

[42] Deal RB, Henikoff JG, Henikoff S (2010) Genome-wide kinetics of nucleosome turnover determined by metabolic labeling of histones. Science 328: 1161–1164. 20508129 10.1126/science.1186777 PMC2879085

[43] Henikoff S, Henikoff JG, Sakai A, Loeb GB, Ahmad K (2009) Genome-wide profiling of salt fractions maps physical properties of chromatin. Genome Res 19: 460–469. 19088306 10.1101/gr.087619.108 PMC2661814

[44] Bell O, Schwaiger M, Oakeley EJ, Lienert F, Beisel C, et al (2010) Accessibility of the Drosophila genome discriminates PcG repression, H4K16 acetylation and replication timing. Nat Struct Mol Biol 17: 894–900. 20562853 10.1038/nsmb.1825

[45] Thurman RE, Rynes E, Humbert R, Vierstra J, Maurano MT, et al (2012) The accessible chromatin landscape of the human genome. Nature 489: 75–82. 22955617 10.1038/nature11232 PMC3721348

[46] Tolstorukov MY, Goldman JA, Gilbert C, Ogryzko V, Kingston RE, et al (2012) Histone Variant H2A.Bbd Is Associated with Active Transcription and mRNA Processing in Human Cells. Mol Cell 47: 596–607. 22795134 10.1016/j.molcel.2012.06.011 PMC3708478

[47] Schwartz YB, Linder-Basso D, Kharchenko PV, Tolstorukov MY, Kim M, et al.. (2012) Nature and function of insulator protein binding sites in the Drosophila genome. Genome Res. 10.1101/gr.138156.112 PMC3483548 22767387

[48] Karpen GH (1994) Position-effect variegation and the new biology of heterochromatin. Curr Opin Genet Dev 4: 281–291. 8032206 10.1016/s0959-437x(05)80055-3

[49] Girton JR, Johansen KM (2008) Chromatin structure and the regulation of gene expression: the lessons of PEV in Drosophila. Adv Genet 61: 1–43. 18282501 10.1016/S0065-2660(07)00001-6

[50] Li M, Belozerov VE, Cai HN (2010) Modulation of chromatin boundary activities by nucleosome-remodeling activities in Drosophila melanogaster. Mol Cell Biol 30: 1067–1076. 19995906 10.1128/MCB.00183-09 PMC2815568

[51] Chen X, Chen Z, Chen H, Su Z, Yang J, et al (2012) Nucleosomes suppress spontaneous mutations base-specifically in eukaryotes. Science 335: 1235–1238. 22403392 10.1126/science.1217580

[52] Dion MF, Kaplan T, Kim M, Buratowski S, Friedman N, et al (2007) Dynamics of replication-independent histone turnover in budding yeast. Science 315: 1405–1408. 17347438 10.1126/science.1134053

[53] Sakai A, Schwartz BE, Goldstein S, Ahmad K (2009) Transcriptional and developmental functions of the H3.3 histone variant in Drosophila. Curr Biol 19: 1816–1820. 19781938 10.1016/j.cub.2009.09.021 PMC2783816

[54] Kent WJ, Sugnet CW, Furey TS, Roskin KM, Pringle TH, et al (2002) The human genome browser at UCSC. Genome Res 12: 996–1006. 12045153 10.1101/gr.229102 PMC186604

[55] Pauler FM, Sloane MA, Huang R, Regha K, Koerner MV, et al (2009) H3K27me3 forms BLOCs over silent genes and intergenic regions and specifies a histone banding pattern on a mouse autosomal chromosome. Genome Res 19: 221–233. 19047520 10.1101/gr.080861.108 PMC2652204

[56] Barski A, Cuddapah S, Cui K, Roh TY, Schones DE, et al (2007) High-resolution profiling of histone methylations in the human genome. Cell 129: 823–837. 17512414 10.1016/j.cell.2007.05.009

[57] Meyer CA, He HH, Brown M, Liu XS (2011) BINOCh: binding inference from nucleosome occupancy changes. Bioinformatics 27: 1867–1868. 21551136 10.1093/bioinformatics/btr279 PMC3117357

[58] He HH, Meyer CA, Shin H, Bailey ST, Wei G, et al (2010) Nucleosome dynamics define transcriptional enhancers. Nat Genet 42: 343–347. 20208536 10.1038/ng.545 PMC2932437

[59] Crooks GE, Hon G, Chandonia JM, Brenner SE (2004) WebLogo: a sequence logo generator. Genome Res 14: 1188–1190. 15173120 10.1101/gr.849004 PMC419797

