# Supplementary material for: Genome-Wide Identification of Chromatin Transitional Regions Reveals Diverse Mechanisms Defining the Boundary of Facultative Heterochromatin
Source: PLoS One. 2013 Jun 28;8(6):e67156. doi: 10.1371/journal.pone.0067156 (PMC3696093; doi:10.1371/journal.pone.0067156)
Supplement: File S1 — Figures S1–S8 and Tables S1–S2. Figure S1. Construction of empirical positive and negative evaluation datasets. For the optimization of the parameters, we set up the empirical positive and negative evaluation datasets for CTR. For the positive dataset, we took the genomic loci which, (1) show clear H3K27me3 enrichment transition (H3K27me3 tags in the 4 kb region on the enriched and depleted side should be greater than 1.5 fold or less than 25% of genome average, respectively), (2) has a single enrichment region for one of the three known insulator proteins Su(Hw), dCTCF, and BEAF-32 and respective co-factors (CP190 and Mod(mdg4) for Su(Hw), and CP190 for dCTCF and BEAF-32) in the vicinity of the transition. With these criteria, we got 81 positive loci, among which 66, 8 and 7 are bound by BEAF-32, dCTCF and Su(Hw) respectively. For CTR-negative dataset, we used the following criteria, (1) a gene with corresponding genomic region greater than 5 kb and does not overlap with any other gene(s), and (2) the gene should have no alternative splicing, and (3) the average H3K27me3 ChIP-Seq tags in the transcribed region of the gene should be more than two-fold of the whole genome average. We scanned all the annotated genes, 159 of them went through this unbiased selection for S2 cells. To avoid possible chromatin barriers at TSS (transcription start site) or TES (transcription end site), we took the central 60% regions of each gene to form the negative dataset. Sample regions in positive and negative evaluation datasets. (A) A sample positive region on chromosome 3R. The region has enrichment of DNA-binding insulator protein Su(Hw) and co-factors CP190 and Mod(mdg4), while it does not have enrichment of other two DNA-binding insulator proteins (dCTCF, BEAF-32). In addition, there is dramatic transition in terms of H3K27me3 signal around this region. The dashed line indicates the midpoint of Su(Hw) enriched region, and it was also taken as the coordinate of this positive regi [file pone.0067156.s001.doc]

**Figure S1.** Construction of empirical positive and negative evaluation datasets.

For the optimization of the parameters, we set up the empirical positive and negative evaluation datasets for CTR. For the positive dataset, we took the genomic loci which, (1) show clear H3K27me3 enrichment transition (H3K27me3 tags in the 4kb region on the enriched and depleted side should be greater than 1.5 fold or less than 25% of genome average, respectively), (2) has a single enrichment region for one of the three known insulator proteins Su(Hw), dCTCF, and BEAF-32 and respective co-factors (CP190 and Mod(mdg4) for Su(Hw), and CP190 for dCTCF and BEAF-32) in the vicinity of the transition. With these criteria, we got 81 positive loci, among which 66, 8 and 7 are bound by BEAF-32, dCTCF and Su(Hw) respectively.

For CTR-negative dataset, we used the following criteria, (1) a gene with corresponding genomic region greater than 5kb and does not overlap with any other gene(s), and (2) the gene should have no alternative splicing, and (3) the average H3K27me3 ChIP-Seq tags in the transcribed region of the gene should be more than two-fold of the whole genome average. We scanned all the annotated genes, 159 of them went through this unbiased selection for S2 cells. To avoid possible chromatin barriers at TSS (transcription start site) or TES (transcription end site), we took the central 60% regions of each gene to form the negative dataset.

Sample regions in positive and negative evaluation datasets. (A) A sample positive region on chromosome 3R. The region has enrichment of DNA-binding insulator protein Su(Hw) and co-factors CP190 and Mod(mdg4), while it does not have enrichment of other two DNA-binding insulator proteins (dCTCF, BEAF-32). In addition, there is dramatic transition in terms of H3K27me3 signal around this region. The dashed line indicates the midpoint of Su(Hw) enriched region, and it was also taken as the coordinate of this positive region. (B) A sample negative region on chromosome 2L. The entire transcribed region of the gene *CG4341* is covered by high level of H3K27me3. The central 60% of this gene (the region between two dashed lines) was taken as a negative region.


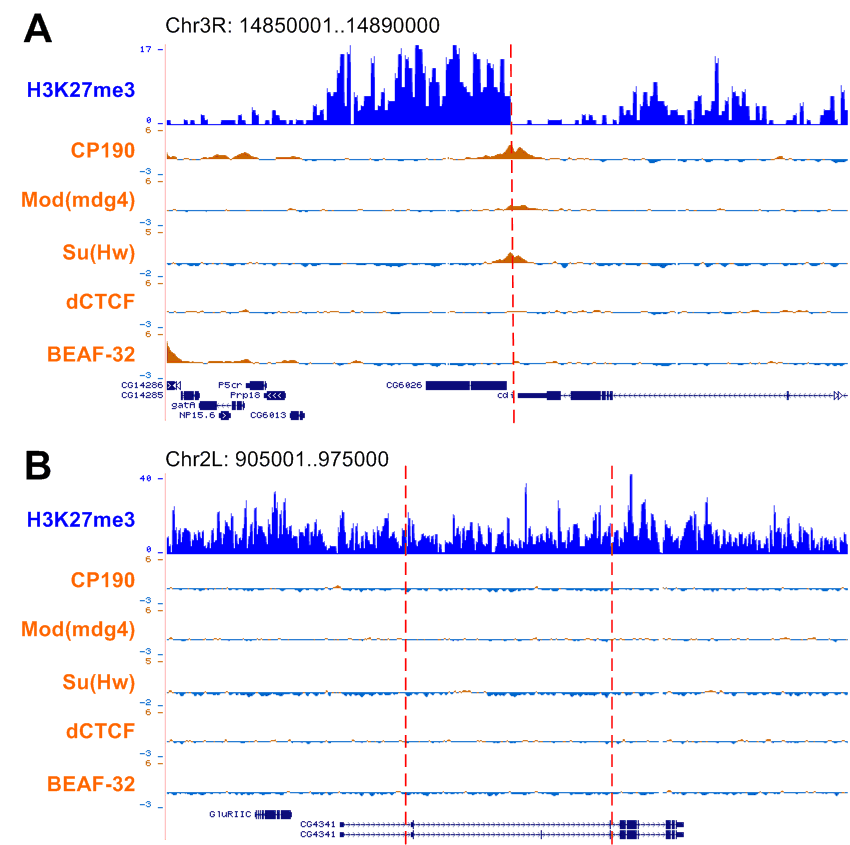


**Figure S2.**  Comparison of CTRICS with SICER and RSEG. (A) The Venn diagram shows the number of CTRs (predicted by CTRICS) that are overlapping with the chromatin boundaries predicted by the other two methods in S2 cells. (B) False-positive and false-negative rates for different methods based on the empirical evaluation datasets. (C) A region shows several examples of CTRs predicted by CTRICS (red bar), H3K27me3 boundaries predicted by RSEG (grey bar), and H3K27me3 domains predicted by SICER. RSEG missed the three boundaries shown in the blue dashed block, which can be corroborated with RNA-Seq and H3K4me3 ChIP-chip data.


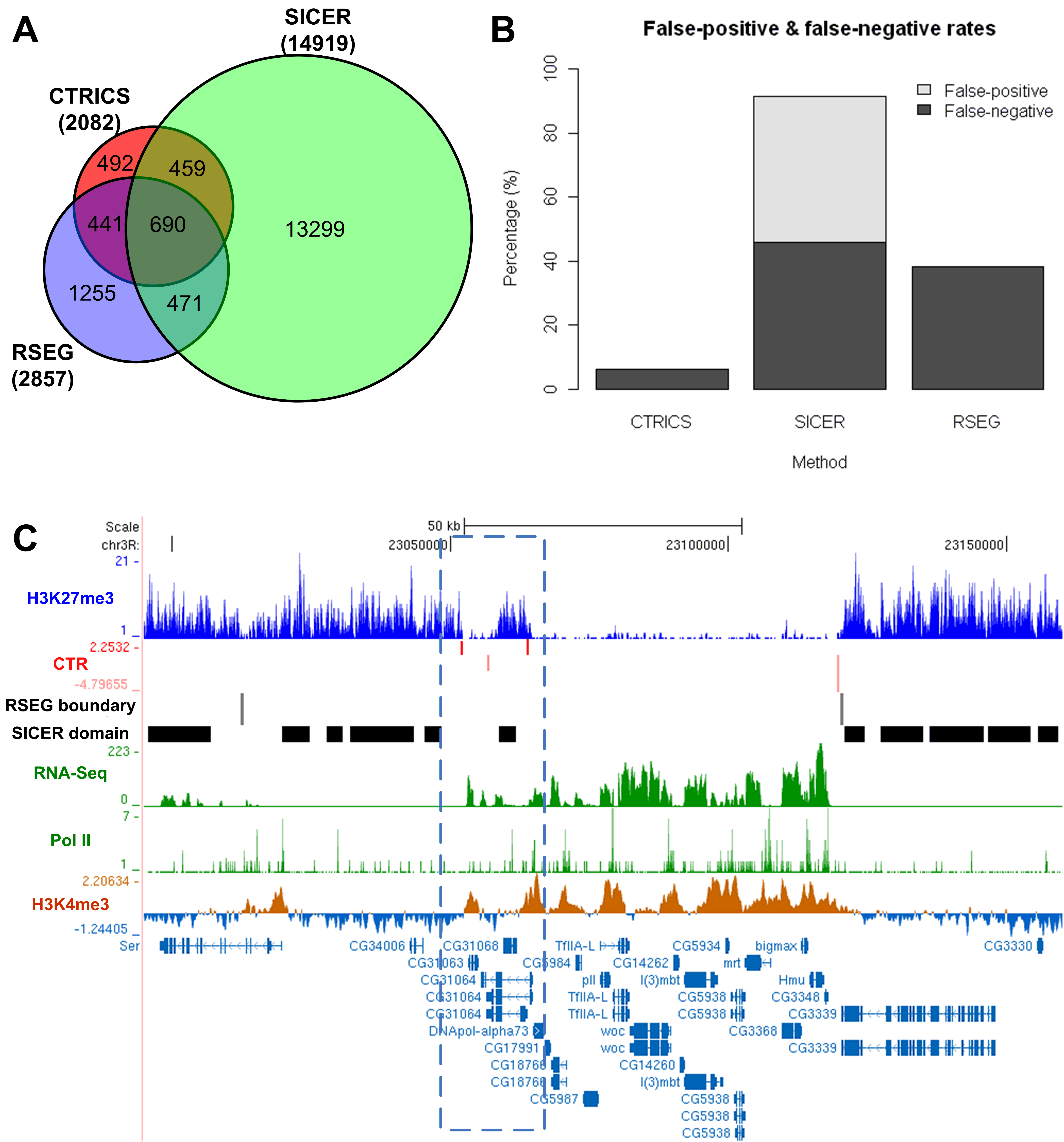


**Figure S3.** Sequencing depth analysis. In order to test if the H3K27me3 ChIP-Seq dataset has reached saturation status and if sequencing depth has any influence on the CTR prediction, we conducted the sequencing depth analysis. We first randomly extracted a series of subsamples (10%, 20%, 30%, and so on until 90% of the original tags) from the H3K27me3 ChIP-Seq dataset without replacement. We then identified chromatin transitional regions in each subsample using CTRICS with default parameters. The x-axis of the plot represents the percentage of subsample tags compared to the total tags (~2.8 x10^6^), and y-axis indicates the number of CTRs identified.


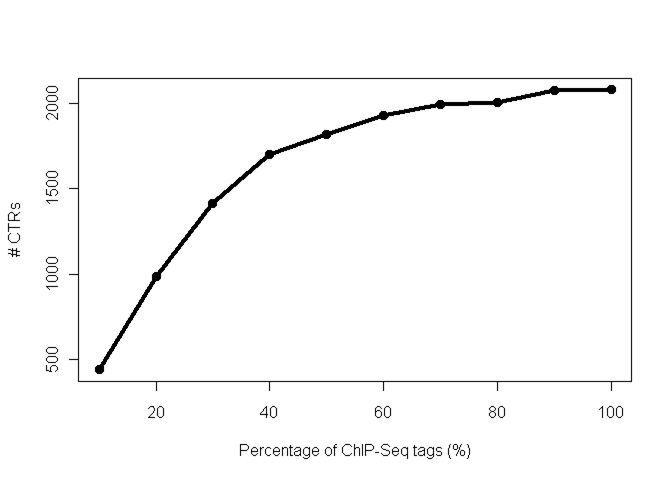


**Figure S4.** Principal component analysis of CTRs based on association with the 15 proteins. (A) Percentage of total variance accounted for by individual principal components. (B) Two-dimensional projections onto the first three principal components. Different colors of the dots represent different groups of CTRs corresponding to the groups shown in the hierarchical clustering result (Figure 3A).


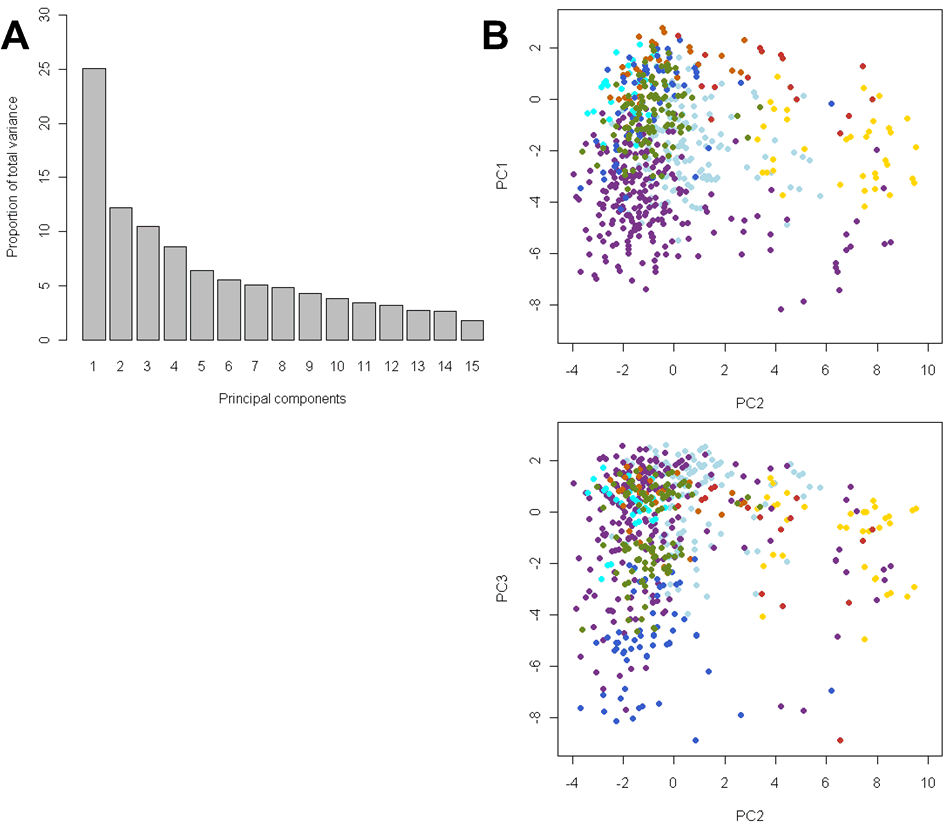


**Figure S5.** Genomic distribution of CTRs. The average intensities of RNA polymerase II (A) and H3K4me3 (B) around individual groups of CTR. (C) The distribution of CTRs in each group.

**
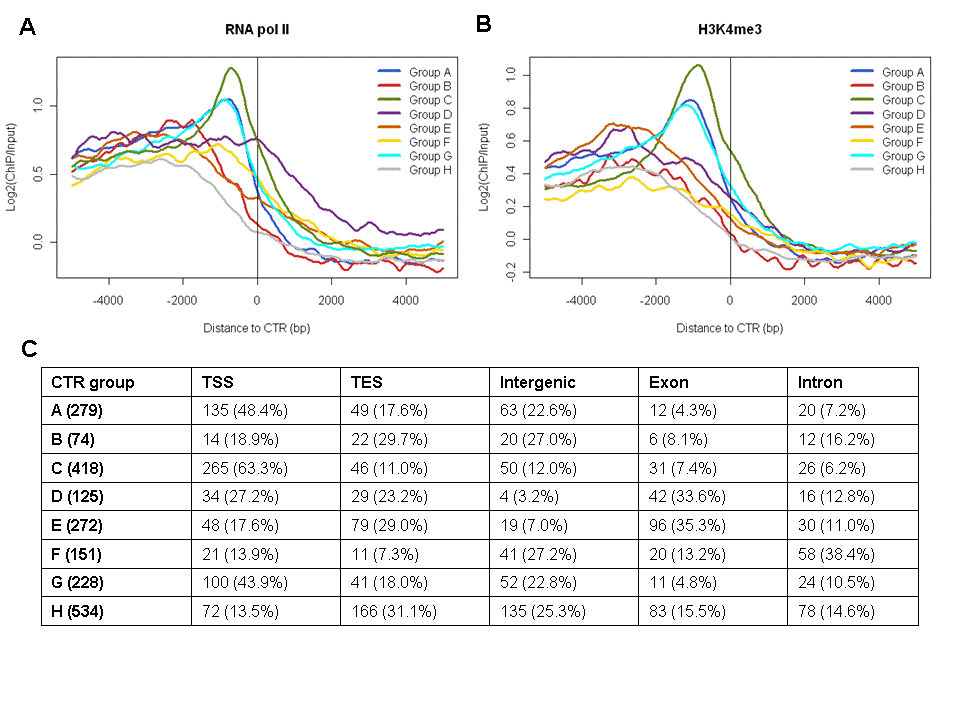
**

**Figure S6.** An example of 2 CTRs (red bars) predicted by CTRICS in human HeLa cells. The panel below CTR shows H3K27me3 domains predicted in Cuddapah *et al.* 2009. CTRICS identifies the boundaries with a significant drop of H3K27me3 level, but ignores the boundaries with minor changes in H3K27me3 signal.


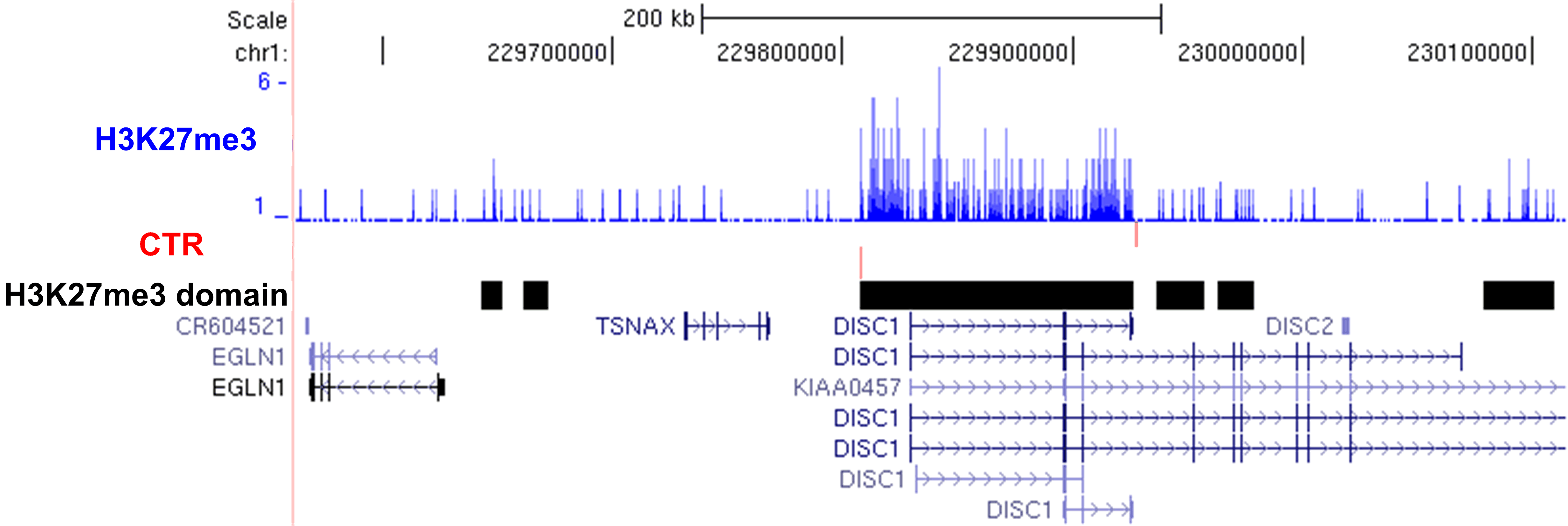


**Figure S7.**  Binding patterns of co-factors in CTR-associated vs. euchromatic binding sites of insulator proteins. Binding patterns of insulator proteins (A) and their co-factors (B) around CTR-associated (solid curve), heterochromatic (dotted curve) and euchromatic (break curve) binding sites in *Drosophila* S2 cells. For CTR-associated binding sites, negative and positive distances denote euchromatic and heterochromatic side.


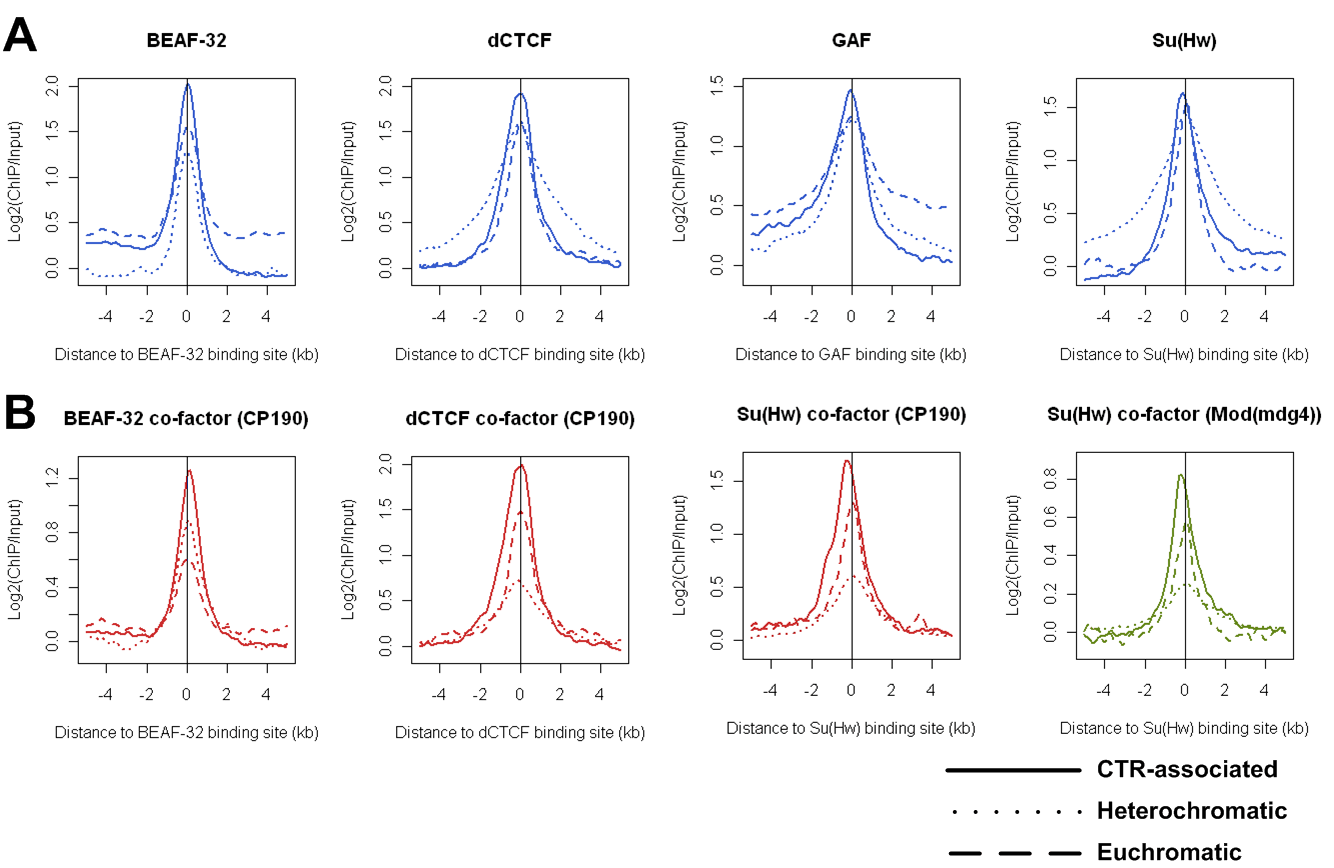


**Figure S8.** Flowchart of CTRICS. The green characters are the parameters needed in each step.


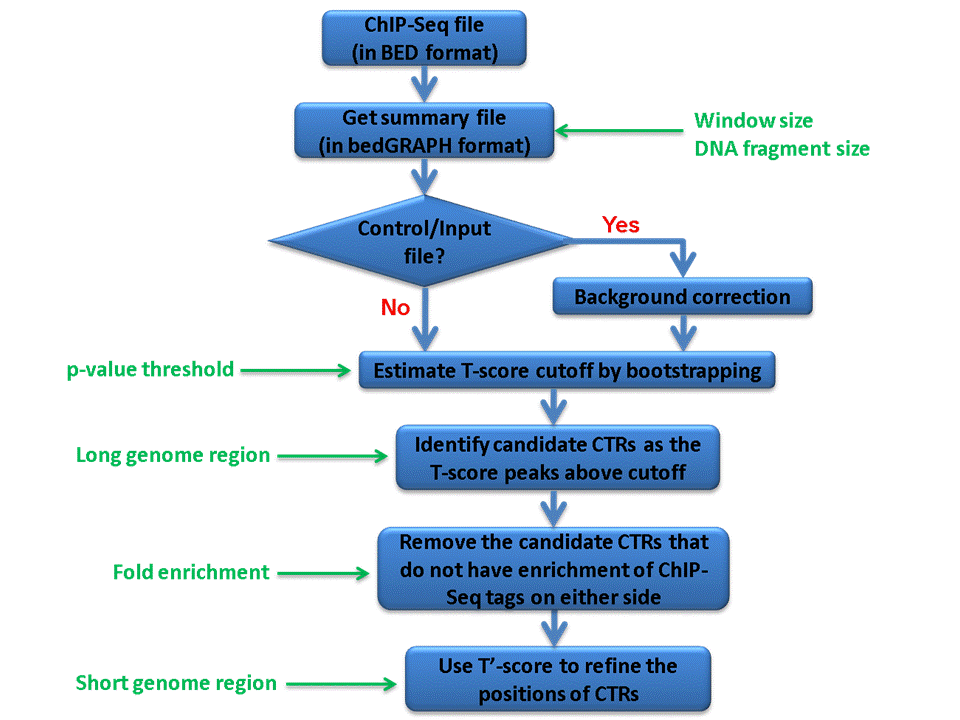


**Table S1.** The list of ChIP-Chip profiles used in the clustering analysis.

| **Protein** | **modENCODE Title** | **DCCid** | **Public Release Date** | **Antibody** | **Cell line** | **Platform** |
| --- | --- | --- | --- | --- | --- | --- |
| Su(Hw) | Su(Hw)-VC.S2 | modENCODE_331 | 10/11/2009 | Su(Hw)-VC | S2 | Affymetrix Drosophila Tiling Arrays v2.0R |
| Mod(mdg4) | mod2.2-VC.S2 | modENCODE_2674 | 02/15/2010 | mod2.2-VC | S2 | Affymetrix Drosophila Tiling Arrays v2.0R |
| dCTCF | CTCF-VC.S2 | modENCODE_283 | 10/11/2009 | CTCF-VC | S2 | Affymetrix Drosophila Tiling Arrays v2.0R |
| GAF | GAF.S2 | modENCODE_285 | 10/11/2009 | GAF | S2 | Affymetrix Drosophila Tiling Arrays v2.0R |
| dMi-2 | dMi-2_Q2626.S2 | modENCODE_926 | 10/11/2009 | dMi-2_Q2626 | S2 | Affymetrix Drosophila Tiling Arrays v2.0R |
| SPT16 | SPT16_Q2583.S2 | modENCODE_3058 | 09/27/2010 | SPT16_Q2583 | S2 | Affymetrix Drosophila Tiling Arrays v2.0R |
| MRG15 | MRG15_Q2481.S2 | modENCODE_3047 | 09/27/2010 | MRG15_Q2481 | S2 | Affymetrix Drosophila Tiling Arrays v2.0R |
| JIL1 | JIL1_Q3433.S2 | modENCODE_945 | 10/12/2009 | JIL1_Q3433 | S2 | Affymetrix Drosophila Tiling Arrays v2.0R |
| RNAPolII | RNA pol II (ALG).S2 | modENCODE_329 | 10/11/2009 | RNA pol II (ALG) | S2 | Affymetrix Drosophila Tiling Arrays v2.0R |
| BRE1 | BRE1_Q2539.S2 | modENCODE_923 | 10/11/2009 | BRE1_Q2539 | S2 | Affymetrix Drosophila Tiling Arrays v2.0R |
| CP190 | CP190-VC.S2 | modENCODE_280 | 10/11/2009 | CP190-VC | S2 | Affymetrix Drosophila Tiling Arrays v2.0R |
| NURF301 | NURF301_Q2602.S2 | modENCODE_947 | 10/12/2009 | NURF301_Q2602 | S2 | Affymetrix Drosophila Tiling Arrays v2.0R |
| Chriz | Chro(Chriz)BR.S2 | modENCODE_278 | 10/11/2009 | Chro(Chriz)BR | S2 | Affymetrix Drosophila Tiling Arrays v2.0R |
| BEAF | BEAF-HB.S2 | modENCODE_274 | 10/11/2009 | BEAF-HB | S2 | Affymetrix Drosophila Tiling Arrays v2.0R |
| WDS | WDS_Q2691.S2 | modENCODE_953 | 10/12/2009 | WDS_Q2691 | S2 | Affymetrix Drosophila Tiling Arrays v2.0R |

**Table S2.** The list of datasets used in this study.

| **Name of dataset** | **Cell line** | **Platform** | **GEO accession #** |
| --- | --- | --- | --- |
| H3K27me3 | S2 | ChIP-Seq | GSM480157 |
| Gene expression | S2 | RNA-Seq | GSM480160 |
| H3K4me1 | S2 | ChIP-Chip | GSE20786 |
| H3K4me2 | S2 | ChIP-Chip | GSE20838 |
| H3K4me3 | S2 | ChIP-Chip | GSE20787 |
| H3K9ac | S2 | ChIP-Chip | GSE20790 |
| H3K9me3 | S2 | ChIP-Chip | GSE20794 |
| H3K27ac | S2 | ChIP-Chip | GSE20779 |
| H3K27me3 | S2 | ChIP-Chip | GSE20781 |
| RNA Polymerase II | S2 | ChIP-Seq | GSM480159 |
| H3.3 (low salt) | S2 | ChIP-Chip | GSM333869 |
| H3.3 (high salt) | S2 | ChIP-Chip | GSM333871 |
| Nucleosome density | S2 | MNase-Chip | GSM333835  GSM333840  GSM333844 |
| DNA accessibility | S2 | Methylation footprinting | GSM441282 |
| Nucleosome turnover | S2 | CATCH-IT | GSM494308 |
| H3K27me3 | HeLa | ChIP-Seq | GSM325898 |
| CTCF | HeLa | ChIP-Seq | GSM325897 |
| DNA accessibility | HeLa | DNase-Seq | GSM816643 |
| Nucleosome density | HeLa | MNase-Seq | GSM937970 |
